# Supplementary material for: Mechanical underwater adhesive devices for soft substrates
Source: Nature. 2025 Jul 23;643(8074):1271–80. doi: 10.1038/s41586-025-09304-4 (PMC12310550; doi:10.1038/s41586-025-09304-4)
Supplement: Supplementary file 1 — Supplementary Texts 1–12, Tables 1 and 2, Figs. 1–16 and References. [file 41586_2025_9304_MOESM1_ESM.pdf]

nature portfolio

https://doi.org/10.1038/s41586-025-09304-4

Supplementary information

Mechanical underwater adhesive devices for soft substrates

In the format provided by the authors and unedited

SUPPLEMENTARY INFORMATION

Mechanical Underwater Adhesive Devices for Soft Substrates

Ziliang Kang[1,2,3], Johanna A. Gomez[1,†], Alisa MeiShan Ross[2,†], Ameya R. Kirtane[2,3,4], Ming Zhao[1,2], Yubin Cai[5], Fu Xing Chen[3], Corona L. Chen[3], Isaac Diaz Becdach[3,6], Rajib Dey[1,2], Andrei Russel Ismael[2], Injoo Moon[3], Yiyuan Yang[1], Benjamin N. Muller[1,2,6], Mehmet Girayhan Say[1], Andrew Pettinari[1,3], Jason Kobrin[1], Joshua Morimoto[3], Ted Smierciak[3], Aaron Lopes[2,3], Ayten Ebru Erdogan[2], Matt Murphy[1], Niora Fabian[1,3,7], Ashley Guevara[3], Benedict Laidlaw[1,3], Kailyn Schmidt[1], Alison M. Hayward[1,2,3,7], Alexandra H. Techet[1], Christopher P. Kenaley[8], Giovanni Traverso[1,2,3,6,*]

1 Department of Mechanical Engineering, Massachusetts Institute of Technology, Cambridge, MA 02139, USA.

2 Division of Gastroenterology, Hepatology and Endoscopy, Brigham and Women's Hospital, Harvard Medical School, Boston, MA 02115, USA.

3 David H. Koch Institute for Integrative Cancer Research, Massachusetts Institute of Technology, Cambridge, MA 02139, USA.

4 Department of Pharmaceutics, University of Minnesota, Minneapolis, MN 55455, USA.

5 Program in Media Arts and Sciences, Massachusetts Institute of Technology, Cambridge, MA 02139, USA.

6 Broad Institute of MIT and Harvard, Cambridge, MA 02139, USA

7 Division of Comparative Medicine, Massachusetts Institute of Technology, Cambridge, MA 02139, USA.

8 Department of Biology, Boston College, Chestnut Hill, MA 02467, USA.

†These authors contributed equally

*Please address correspondence to cgt20@mit.edu, ctraverso@bwh.harvard.edu

This PDF file includes:

Supplementary Text
Supplementary Tables. S1 to S2
Supplementary Figures. S1 to S16
Supplementary References

Other Supplementary Materials for this manuscript include the following:

Supplementary Videos. S1 to S9

S1. Coupled Eulerian-Lagrangian (CEL) technique and relative vacuum ratio

The CEL technique is a powerful tool for analyzing solid-fluid interactions (i.e. disk-water and tissue-water interactions) alongside solid-solid interactions (i.e. disk-tissue interactions) simultaneously. This method enables the simulation of liquid flow through Eulerian finite elements, which may not always be entirely filled with material but can be partially or completely void. At the contact surface, the Eulerian elements are coupled with Lagrangian finite elements, which define the deformation of solid materials [1]. Unlike classical fluid element analysis using finite volume methods—which assume a finite volume of fluid, preventing complete water expulsion at the solid-solid contact interface — CEL provides a more realistic validation of hydrodynamic behavior of the adhesive disk. It accounts for the dynamic morphing of the disk and the expulsion of water, enabling a comprehensive analysis of disk-tissue-water interactions when adhering to stomach tissue submerged in water.

To differentiate the hydrodynamic behaviors modeled by finite element analysis for various configurations of remora adhesive disk mimicries, we used the relative vacuum ratio V_r to evaluate the amount of water expelled relative to the theoretical maximum volume of water that can be expelled in the disk during underwater adhesion for each case: an unfurled disk, a furled disk, and a benchmark one-piece disk. Given the complexity of accurately calculating the volume of water retained in the various deformation states of the remora adhesive disk mimicries, we employed free image processing software (Inkscape) to measure the precise area of water held in the cross profile of each configuration. We then calculated the relative vacuum ratio V_r as follow,

$$V_r = \frac{\sum_{n=t_0}^{t_{end}} |A_n - A_{n-1}|}{A_{Initial}} \quad (S.1)$$

where $A_{Initial}$ represents the initial cross-sectional area of the disk mimicry before any deformation, indicating the theoretical maximum amount of water that can be expelled. $|A_n - A_{n-1}|$ measures the cumulative amount of water expelled, beginning from the moment t_0 when the disk mimicry contacts the stomach tissue, until t_{end} when the disk can no longer be pressed to expel water.

S2. Set up of mechanical adhesion test

We recognized that researchers from diverse backgrounds use varying nomenclatures and test setup when evaluating adhesive performance. Accordingly, the mechanical testing method detailed in the Methods section was designed to be balanced and comprehensive, ensuring fair comparisons across different adhesive solutions while considering variations in test setups. Specifically, the adhesion strength values for Carbopol and EDC-NHS tested in this manuscript were expected to be lower than reported literature [2, 3]. Further, Silbione, as referenced [4], were expected fail to form adhesion on moist tissue surfaces. As discussed in the main manuscript, polymeric adhesives typically exhibit increased strength following a prolonged, pressurized pre-adhesion process (5-30 minutes) [2]. To ensure a fair comparison, we limited the pre-adhesion pressurization for NHS-EDC and Carbopol hydrogels to 3 minutes, since MUSAS achieves instant mechanical adhesion. Furthermore, mechanical adhesion tests for polymeric adhesives often involve trimming tissue to a thickness of 1-2 mm to match the thickness of the polymeric adhesive [2, 3]. However, stomach and other organ tissues are significantly thicker (8-

10 mm) [5], with dynamic sliding between the serosal and mucosal layers that affects adhesion performance (Supp. Fig. S5). Additionally, polymeric adhesive tests are typically conducted on the serosal or epidermal layers, requiring surface washout and the removal of liquid accumulation from wet surfaces [2, 3]. Besides, adhesion tests were often conducted 24 hours post-application to account for equilibrium swelling of the polymeric adhesive [3]. However, in such cases, post-mortem autolysis and tissue decomposition may occur, and adhesion strength is often measured at deeper serosal or muscular layers. In contrast, the mechanical adhesion testing in our study was performed on freshly harvested tissue (< 1-hour post-euthanasia) without surface washout, tissue trimming, or liquid removal. The loading rate of the mechanical test considered the ASME tissue adhesion standards that were adopted by the polymeric adhesive community. Soft substrates used in test were only partially secured, with the four corners of the tissue squares glued to the holder to allow for natural sliding and dynamic morphing. These factors account for the fair yet expectedly lower adhesion strength of polymeric adhesives compared to previously reported data.

S3. Extended discussion on effect of lamella orientation angle and number of rows on MUSAS adhesion to soft substrates

Adhesion to soft substrates is impacted by several critical factors including roughness, intactness, stiffness, and dynamic morphing. Indeed, dynamic sliding and surface morphing often occur in softer substrates of stiffness ranging from 10 kPa to 100 kPa, such as sliding between the serosal and mucosal layers (Supp. Fig. S5). Additionally, the roughness of soft substrates is often non-uniform; for instance, a surface with an areal average roughness of 200 μm can exhibit significantly bumpy or pitted regions with maximum roughness up to 600 μm, which brings significant challenges for conformal vacuum-based water-tight seal (Fig. 3g). Moreover, in real-world scenarios, soft substrates generally lack rigid support, making them susceptible to the side effects of dynamic morphing in maintaining adhesion, requiring further evaluation such as *in vivo* invalidation, which was overlooked by prior research.

Indeed, we found that the understanding derived from past research cannot be directly applied to remora-inspired adhesion to soft substrates, such that shear frictional performance is not sensitive to lamella contact (pitching) angle but the spinule lengths, due to the dynamic morphing of soft substrates (Supp. Fig. S6). Specifically, we aim to understand whether a particular angle of lamella orientation and the number of rows contribute to adhesion. Hence, we added five designs of lamella orientation fixed with a particular tilted angle, considering different numbers of rows of lamellae, including 30-degree dominated orientation with 4 rows of lamellae, 30-degree dominated orientation with 6 rows of lamellae, 30-degree dominated orientation with 8 rows of lamellae, 15-degree dominated orientation with 8 rows of lamellae, and 45-degree dominated orientation with 8 rows of lamellae, comparing with the tilted-dominant orientation design (0-15-30-45-degree orientation with 8 rows of lamella), on soft substrates of different stiffness and roughness. These results further highlighted the importance of understanding the impact of the lamella orientation angle and number of rows on the adhesion to soft substrates with varying stiffness, as well as on retention performance (Supp. Fig. 7). While the optimal tilt-dominant orientation design was confirmed to outperform the other configurations, a clear trend emerged showing that adhesion on rough surfaces is always weaker than on smooth surfaces. This trend is particularly evident in adhesion performance on soft substrates with similar stiffness but significantly different roughness (bis-tris gel vs. porous tough hydrogel). It is worth noting that in rare cases, although the trend is preserved across all designs, the statistics were

insignificant. This is due to measurement disparity caused by the nonuniformity and dynamic morphing of the gels, especially considering their nonuniform fragility and porosity. Additionally, adhesion on stiffer substrates is clearly stronger than on softer substrates with similar roughness (porous tough hydrogel vs. SEBS), due to less dynamic morphing of the stiffer substrates, which helps preserve the adhesion seal of the devices. Interestingly, we also observed shear sliding behaviors on all 15-, 30-, and 45-degree dominated orientations (Supp. Fig. S7), similar to what we previously observed in the parallel-dominant design (Ext. Fig. 2).

To further strengthen the scientific evaluation and eliminate confounding variables, we conducted a new set of controlled experiments using artificial substrates designed to independently isolate the effects of roughness and stiffness. Specifically, we fabricated two sets of silicone substrates: one with identical surface roughness but varying stiffness (Zhermack Elite Double 8 vs. Double 32 silicone rubber), and one with identical stiffness but varying roughness (smooth vs. roughened Zhermack Elite Double 8). We tested representative designs on these artificial substrates, including the 30-degree-dominated, 8-row design and the tilt-dominant, 8-row design—the top two performers in mechanical testing—as well as the 30-degree-dominated, 4-row design (Supp. Fig. S8). The results reaffirmed a clear trend: adhesion was consistently weaker on rougher surfaces compared to smooth surfaces when stiffness remained constant. Additionally, adhesion to softer substrates was consistently weaker than on stiffer substrates when surface roughness was controlled.

All these findings revealed that improved adhesion performance on soft substrates does not stem from a particular angle of lamella orientation but rather from the variation in lamella orientation. This reinforces our previous conclusion that a particular contact angle of lamellae does not play a significant role in adhesion on soft substrates, due to the contact variation caused by the dynamic morphing and non-uniformity of soft substrates. Furthermore, a smaller number of rows significantly weakens adhesion performance due to the lack of independent co-adhesion resulting from a limited number of individual compartments.

A comprehensive analysis elucidating the performance variations across different designs observed in the aforementioned mechanical studies (Supp. Fig. S7) is necessary. Meanwhile, the focus of this study on adhesion to soft substrates introduced an additional layer of complexity in experimentally characterizing the volume change of the devices compared to previous suction-based adhesion studies, since the most commonly adopted approaches, such as frustrated total internal reflection (FTIR) imaging, require fully transparent hard substrates (glass), for surface analysis. To overcome this obstacle, we designed a new approach by combining micro-CT and particle image velocimetry (PIV) to comprehensively study the volume change of MUSAS with different numbers of rows and lamella orientations adhering to soft substrates with various stiffness and roughness.

S3.1 Micro-CT imaging of internal volume changes of different designs

We imaged the internal volume of different designs adhered to bis-tris gel (stiffness: 9 kPa, areal average roughness Sa: 2.592 μm), porous tough hydrogel (stiffness: 26 kPa, Sa: 19.118 μm), and pig stomach tissue (stiffness: 76 kPa, Sa: 85.164 μm) in an underwater environment through a SkyScan 1276 micro-CT (Bruker) with SkyScan 1276 application software. To comprehensively evaluate the internal volume change and avoid measurement bias, we calculated both the relative vacuum ratio and the relative compression ratio (Ext. Fig. 3c-3d). In accordance with the mechanical testing results (Supp. Fig. 7), the tilt-dominant design outperformed all the others,

and an increased number of rows consistently enhanced independent compartmental sealing, reinforcing our previous findings (Fig. 2j). Notably, a clear trend was once again confirmed: adhesion on rougher surfaces was always weaker than on smooth surfaces (bis-tris gel vs. porous tough hydrogel). Additionally, adhesion on stiffer substrates was stronger than on softer substrates with similar roughness (porous tough hydrogel vs. pig stomach tissue). Micro-CT analysis elucidated that the superior performance of the tilt-dominant design with variational lamella orientation resulted from well-rounded mechanical interlocking in all directions, extending below the contact surface to secure suction-based adhesion. This adaptation conformed to the dynamic, non-homogeneous morphing of the substrates, such that the individual adhesive compartments of the optimal tilt-dominant MUSAS were better vacuumized and filled with bis-tris gel and stomach tissue (Ext. Fig. 3e). Furthermore, micro-CT images explained why the 30 deg-dominated design often outperformed the 15 deg-dominated and 45 deg-dominated designs in mechanical testing (Ext. Fig. 3c, 3d), such that the 30 deg-dominated design demonstrated better interlocking than the others, measured by the relative vacuum ratio and the relative compression ratio, yet still lacked sufficient angular variation comparing to the tilt-dominant design (Ext. Fig. 3e). Note that the relative vacuum ratio measurement for the 45 deg-dominated design may be biased, as the real value could be lower due to non-vacuum space concealed by its highly tilted lamella orientation (Ext. Fig. 3e), which necessitates evaluation of both the relative vacuum ratio and the relative compression ratio.

S3.2 Particle image velocimetry (PIV) evaluation of the hydrodynamic performance of different designs

We further conducted PIV tests to understand the hydrodynamic performance of different designs. Specifically, we selected representative designs evaluated through micro-CT testing, along with the benchmark parallel-angled design, to assess their water expulsion performance at a constant sinking speed of approximately 30 mm/min for adhesion to a bottom substrate (Ext. Fig. 4). Nanoparticles of 50 μm diameter (Dantec) were added to the water at a concentration of 3-10 particles per pixel for velocimetry. Their flow trajectories were illuminated using a shuttered continuous-wave (CW) laser (LaVision), recorded at 250 fps with a high-speed camera (Edgertronic), and processed using the MATLAB R2022a (Mathworks) toolbox PIVlab [6] within a region of interest (ROI) of 330 × 480 pixels. High-speed camera images were pre-processed with contrast-limited adaptive histogram equalization (CLAHE), intensity capping, denoising, and automatic contrast stretching. PIV settings included three passes, using interrogation areas of 64 × 64, 32 × 32, and 16 × 16 pixels with 50% overlap. Bottom-view instantaneous velocity fields reveal that designs with fewer lamella rows exhibit minimal water expulsion, as observed in the 4-row configuration (Ext. Fig. 4a). This explains their inadequate internal volume change during adhesion, observed in the mechanical and micro-CT testing. As the number of lamella rows increases, unsubstantial angle tilting tends to facilitate unidirectional water expulsion, such that the 15-degree-dominated design performing nearly identically to the parallel-angled configuration (Ext. Fig. 4b, 4c). In contrast, designs with significant angle tilting induce multidirectional water expulsion, as evidenced by the strong local convergence and divergence of flow vectors, indicating out-of-plane (3D) flow (Ext. Figs. 4d–4f). However, water expulsion in designs dominated by 30-degree and 45-degree angles is often uneven and accompanied by significant air bubble formation in specific regions (Ext. Figs. 4d, 4e). Among the tested configurations, the tilt-dominant design with substantial lamella angle variation, demonstrates superior efficiency in achieving homogeneous, multidirectional water expulsion

(Ext. Fig. 4f). Combined with its well-rounded, omnidirectional mechanical interlocking, this design further confirms that improved adhesion performance is driven by variations in lamella orientation angles rather than a strong preference for dominance of a particular lamella orientation angle.

S4. Mechanical reliability and MUSAS retention under dynamic interference

S4.1 In vitro evaluation

We further conducted *in vitro* dynamic inference studies to compare the retention performance of the 30-degree dominated, 8-rows design with the tilt-dominant, 8-row designs, which are the top two best performers in the mechanical test (Ext. Fig. 5). Specifically, different designs were adhered to stomach tissue phantoms freely floating in simulated gastric fluid (pH = 1.5), shaken in a 37°C incubator (Ext. Figs. 5a–5c, Supp. Video S6). The results once again confirm the tilt-dominant design as an outperformer, such that it can maintain on the tissue phantom for more than 12 days.

S4.2 In vivo evaluation

In addition to the aforementioned *in vitro* dynamic interference studies, the mechanical stability of MUSAS was further validated *in vivo* through endoscopic interference studies. Supp. Video S6 serves as an example, demonstrating the adhesion stability of MUSAS when subjected to touching, pushing and shaking interference. Further mechanical stability of MUSAS validated *in vivo* in a dynamic, unstructured and often unpredictable environment was discussed in the main manuscript. Specifically, we tested GI retention in pigs (> 9) and body surface retention in fish (> 6) under survival conditions, referring to monitoring the animals without interfering with their normal feeding, resting, or behaviors (Fig. 4, Ext. Fig. 5). This approach ensured evaluation under dynamic conditions, including complex movements, peristalsis, variable fluid flow, and mechanical forces.

S5. Minimized preloading requirements for MUSAS

We characterized adhesion performance of MUSAS under various preloading conditions, using underwater adhesion on a stomach tissue phantom as an example (Supp. Fig. S9). The results indicate that a preload as low as 0.05 N enables MUSAS adhesion, while 0.2 N is sufficient to achieve near-optimal adhesion performance. These findings confirm the successful minimization of MUSAS preloading requirements through optimal lamella orientation and disk furling, highlighting its unique self-adhesion capability driven by GI tract contractions (Fig. 4e, Supp. Video S7).

S6. Technical rigor and translational relevance of animal models

Unlike studies that merely propose conceptual applications, each MUSAS-enabled application underwent rigorous, independent *in vitro*, *ex vivo*, and *in vivo* validation, employing gold-standard animal models that offer advance translational relevance. In this study, we conducted survival studies under dynamic and natural conditions, ensuring minimal interference with normal behaviors such as feeding, resting, and swimming. When the use of real tissue is restricted due to animal welfare considerations in experimental studies, stomach tissue phantom serves as a realistic tissue simulator. In this study, stomach tissue phantom was developed by the LifeLike Biotissue company, which is one of the leading providers of tissue phantoms in North

America and is widely used by major biomedical companies, including Medtronic, Johnson & Johnson, Edwards Lifesciences, and Boston Scientific.

S6.1 Swine study

Our study employs highly complex and translationally relevant animal models, particularly the swine model, which closely mirrors human anatomy, physiology, immunology, and genomics. Swine studies often serve as the final preclinical step before clinical trials but introduce significantly greater validation complexity and failure risk compared to small-animal models [7]. Specifically, MUSAS were tested in over 58 pigs in three years, as discussed in the main manuscript under survival conditions. The pigs were fed and monitored daily in the morning and afternoon with a laboratory mini-pig grower diet laboratory mini-pig grower diet, 5081, along with midday snacks of fruits and vegetables. Survival tests were conducted without interfering with pigs' normal feeding, resting, or behaviors.

S6.2 Fish study

Remora (*Echeneis naucrates*) and tilapia (*Oreochromis niloticus*) were single-housed in a 40-gallon tank (91.44 cm L × 43.18 cm W × 45.72 cm H) for the fish study. The remora was kept in saltwater at 28 °C, while the tilapia was maintained in freshwater at room temperature (20 °C). Additionally, a gourami (*Osphronemidae*) was housed separately in a 2-gallon tank (20 cm L × 20 cm W × 20 cm H) with freshwater at 27 °C.

The experimental setup for mechanical testing of remoras (Echeneis naucrates) is shown in Supplementary Fig. 2. For live remora studies, a Series 4 force gauge (Mark-10) equipped with Mesur Lite v2.0.0 software was used. For testing euthanized specimens, a 5944 universal testing system (Instron) with Bluehill v3.11 software was employed. Details of the Instron setup are provided in the Methods section.

For the long-term retention study of MUSAS adhering on tilapia skin, two action cameras (GoPro) were used to capture the tilapia's movements every hour, allowing for the monitoring of MUSAS detachment. Adhesion of MUSAS on tilapia was instant, and the deployment process can be seen in Supp. Video S8.

Note that Fig. 1 and its corresponding section in the main manuscript focus exclusively on studies of the remora itself. Specifically, the experiments related to Figs. 1g and 1h are designed to characterize the mechanical performance of a live remora's adhesion to soft substrates. If such tests cannot be conducted on a live remora, they are supplemented with tests on euthanized specimens. That being said, the use of real pig stomach tissue (RST) is prohibited in a live remora experiment due to animal welfare concerns and does not align with the policies of the Massachusetts Institute of Technology's Committee on Animal Care and Boston College's Institutional Animal Care and Use Committee. This decision also accounts for biohazard risks and the long-term interaction of alive remoras with soft substrates. Nevertheless, the data provided is comprehensive enough for evaluation, given the multiple substrates and scenarios we examined. Additionally, due to current technological limitations, the adhesion of live remoras in the shear direction cannot be tested, as we cannot train them to slide in that particular direction. Nevertheless, it was supplemented by the mechanical test on euthanized remoras. Additionally, adhesion forces measured with live remoras were expected to be slightly lower than those measured with euthanized specimens. This discrepancy arises because the force tester used for

live remoras, which prioritizes animal welfare, has a sampling rate of 0.1s, whereas the universal mechanical tester used for euthanized specimens operates at a much higher 0.4ms sampling rate. Additionally, the larger tank required for housing alive remoras introduces water-induced measurement fluctuations. Despite these challenges, the current experimental setup has been optimized to balance both measurement quality and animal welfare.

S7. Controlled location specific deployment and detachment of MUSAS in the GI tract

S7.1 Controlled location specific deployment of MUSAS in the GI tract

Programmable organ targeting remains a major goal in drug delivery. Many drug formulations, for instance, exhibit optimal absorption in nonacidic environments. Quinidine, a therapeutic used to treat irregular heartbeats, exemplifies this need, requiring precise release in the small intestine [8, 9].

Our close collaboration with physicians and pharmaceutical scientists has directed this study toward a passive-control approach, focusing on three key aspects of drug delivery: 1) miniaturization, preferably to an ingestible size; 2) achieving a high active pharmaceutical ingredient (API) release rate without sacrificing drug loading for motors and controllers; and 3) simple, user-friendly deployment without the need for additional maneuvering equipment or specialized training. This approach contrasts with active-robotics-based strategies, which often depend on large MRI and fluoroscopy systems for active navigation—an aspect that, in our view, limits clinical feasibility due to complex facility and surgical requirements. Here, we demonstrated robust, programmable organ targeting using Eudragit S100, an FDA-approved, commercially-adopted enteric coating polymer for GI organ targeting [10-12]. Specifically, we demonstrate a pH-responsive deployment strategy and showcase how MUSAS can be precisely delivered to the small intestine (Ext. Fig. 6). We utilized the polymethacrylate-based copolymer Eudragit S100, which dissolves in natural to alkaline environments (pH > 7.0). The dip-coating solution was prepared by dissolving Eudragit in ethanol. *In vitro* characterization confirmed that MUSAS encapsulation remains stable for up to 24 hours in simulated gastric fluid, depending on the S100 coating thickness (Ext. Fig. 6a). Additionally, release timing in a simulated intestinal environment can be precisely controlled by adjusting the coating thickness and S100 concentration (Ext. Fig. 6b, 6c). *In vivo* results further demonstrated safe gastric emptying in the stomach and timely deployment in the small intestine (Ext. Fig. 6d). The *in vivo* results confirmed timing and location of release matched the *in vitro* predictions (Ext. Fig. 6b), supporting translational relevance.

Notably, other pH-responsive copolymer materials can be leveraged to enable programmable, targeted delivery of MUSAS to different regions of the GI tract. Future studies may also explore replacing the disk materials of MUSAS with ferromagnetic silicone rubber to enable hand-size magnet-assisted delivery. A detailed discussion has been added to the supplementary information.

S7.2 Controlled location specific detachment of MUSAS in the GI tract

Choices of biodegradable lamella materials to program loss of mechanical interlocking can be used to achieve controllable adhesion and detachment, as discussed in Fig. 4b. We have also added these *in vivo* retention studies of different lamella materials to Fig. 4c and Ext. Fig. 5e. Compared to nitinol lamella with shape memory effects which can stay up to 22 days (Fig. 4c), retention of stainless steel lamellae based devices is about 1 days due to its quick degradation in

acid environment, and superelastic nitinol lamellae-design generally enable retention time up to 4 days (Ext. Fig. 5d, 5e).

S7.3 Clarifying targeting scope: organ vs. wound

Importantly, MUSAS is not designed for wound targeting or hemostatic sealing. While we acknowledge that some adhesives pursue this goal, our platform addresses a distinct and urgent challenge: long-term mucosal retention in a physiologically hostile environment. Note that most oral therapeutics (e.g., for HIV PrEP or metabolic disease) require delivery to a general organ region (e.g., stomach or small intestine) to be effective. Precise lesion targeting ("*in situ* targeting") is outside the scope of this platform and this study. Organ-level targeting is enabled, but *in situ* lesion-specific targeting requires endoscopic diagnosis and delivery, which MUSAS is fully compatible with.

Particularly, it should be noted that wound-site-level targeting is not necessary—or even appropriate—as a benchmark for ingestible therapeutic systems. Most GI diseases addressed by oral therapeutics do not require precise lesion localization. Conditions like metabolic disorders, chronic inflammation (e.g., Crohn's), and infectious disease primarily benefit from organ-level delivery and prolonged mucosal residence, not pinpoint adhesion to a wound [13]. In fact, diffuse and patchy mucosal involvement is the norm in these diseases, making localized targeting both unnecessary and potentially limiting [13]. Conversely, true *in situ* targeting is clinically relevant for GI tumors, which already require endoscopic identification and intervention [14-16]. Imaging-guided robotic navigation inside the GI tract is still experimental, dependent on bulky equipment (MRI, CT, external magnetic fields), and faces major limitations in precision, safety, accessibility, and FDA class III clearance [17-20]. These systems are not deployable in routine care, particularly not for chronic, non-focal diseases.

In contrast, MUSAS is designed as a clinically translatable, passive self-actuation platform, compatible with both oral ingestion and manual endoscopic deployment when precise localization is required (e.g., esophagus, buccal, or upper GI, demonstrated in Fig. 5d, Ext. Fig. 5). This enables real-world integration without specialized infrastructure and delivers the key clinical advantage: long-term residence at mucosal sites without motors, sensors, or power sources.

S8. Design and characterization of MUSAS-enabled ultraminiaturized RFID temperature sensor

S8.1 Design and simulation of the RFID antenna

Designing RFID systems for underwater use presents unique challenges due to the electromagnetic properties of water. The presence of dissolved salts and other materials increases water's conductivity, significantly attenuating radio waves—a phenomenon that is frequency dependent. Our design addresses these challenges by optimizing the antenna and overall system for aquatic operation. The proposed RFID temperature sensor, depicted in Ext. Fig. 7a, has dimensions of 12 mm L × 6 mm W × 1.35 mm H. Both the substrate and superstrate, each 0.0635 mm thick, are fabricated from RT/duroid 6010.2LM laminate (Rogers Corporation). The RFID antenna is a center-symmetrical dipole, fed through the bottom substrate. A ground plane provides moderate shielding from the conducting structures of MUSAS. Additionally, an L-

shaped slot in the ground plane optimizes impedance matching with the Magnus-S RFID temperature sensor chip (Axzon), which has a resistance of 3.5 Ω and a capacitance of 2.58 pF.

Using CST Studio Suite 2022 (SIMULIA), a high-performance 3D electromagnetic (EM) analysis software, we modeled and analyzed the antenna's performance under various conditions, including the presence of water and the attachment of MUSAS (Ext. Fig. 7b). Our antenna design is optimally matched to these conditions, as evidenced by the simulated and measured impedance data presented in Ext. Fig. 7c. The simulated S11 parameter, which quantifies the ratio of input power to radiated power by the passive transponder, indicates excellent impedance matching for the complete assembly, with a minimum value of -38 dB at a resonant frequency of 915 MHz. Notably, the presence of water and the attachment to MUSAS detune the S-parameters, as depicted in Ext. Fig. 7c. The current distribution at a zero-degree phase is presented in Ext. Fig. 7d.

To comprehensively evaluate the backscattering signal in RFID systems, it is essential to assess the antenna's radiation pattern. RFID radiation is not inherently omnidirectional; instead, the strength of the reflected signal from the RFID tag depends on the angle between the reader and the RFID tag, with the tag's information modulated into the signal. Given the antenna's largest dimension (D) of 12 mm and the electromagnetic wavelength in water of approximately 3.7 cm, the far-field range of the antenna can be calculated using the equation $2D^2/\lambda$, resulting in a distance of 0.8 cm. With an operational distance of at least 5 cm, the antenna operates within the far-field range, necessitating an examination of its far-field radiation pattern. In the far field, EM power attenuates radially, following the inverse square law relative to the distance from the antenna. This attenuation means that the radiation pattern does not change shape with increasing distance, making it a critical factor in determining the efficiency and coverage of the RFID system. As shown in Ext. Fig. 7e, the far-field radiation pattern of the proposed RFID antenna exhibits peaks along the azimuth plane, with a null at $\theta = 0°$. Further examination of the 2D cuts in the pattern along the $\phi = 0°$ and $\phi = 90°$ planes, as shown in Ext. Fig. 7f, reveals a distorted donut-shaped pattern. This distortion aligns with the simulated current distribution (Ext. Fig. 7d), where the conducting components of MUSAS share induced current, altering the backside radiation pattern of the dipole antenna. The final directivity of the antenna is 2.91 dBi, with a radiation efficiency of 9%. These results underscore the importance of considering both the antenna's design and its interaction with the device to optimize performance in real-world applications. The use of high-permittivity substrate and superstrate facilitates the miniaturization of the antenna, with potential future work to improve the directivity and efficiency by integrating the MUSAS into the ground plane and enlarging the ground plane footprint.

S8.2 In vitro validation

To ensure accurate and reproducible *in vitro* measurements, we constructed a Faraday cage simulating a water environment for the RFID tags, adhering to the UHF RFID (EPC Class 1 Gen 2) protocol for performance testing. This Faraday cage effectively shields the system from external electromagnetic interference, which is crucial for isolating the antenna's performance characteristics.

Our measurement methodology includes key performance metrics, such as the Received Signal Strength Indicator (RSSI), which provides insights into the signal strength between the RFID tag and the reader. We compared the performance of our RFID temperature sensor to a commercial

Smartrac RFID temperature sensor (Avery Dennison), both utilizing the Axzon Magnus-S tag chip (Ext. Fig. 7g).

A TSL 3166 Bluetooth Rugged UHF RFID Reader (Technology Solutions UK LTD) was placed inside the Faraday cage to read the tags, creating a controlled environment that minimizes external factors affecting signal propagation (Ext. Fig. 7h). We employed the TSL ASCII Software Development Kit – for Android v2.8.0 (Technology Solutions UK LTD) on an Android smartphone for temperature readout (Ext. Fig. 7i).

Ext. Fig. 7j illustrates real-time underwater temperature sensing results from our sensor, compared simultaneously with a commercial RFID temperature tag (Avery Dennison). Our RFID sensor achieved an RSSI range of 3–12 dBm with a power sweep from 15–30 dBm, demonstrating performance comparable to the commercial tag, with minor discrepancies attributed to differences in antenna aperture.

S8.3 Extended in vivo validation

We also tested swimming tilapia with MUSAS-based RFID sensors in a swim tunnel at a water flow of 25 cm/s (~ 1.25 body length (BL)/s) and 45 cm/s (2.25 BL/s), mimicking the characteristic flow of natural, steady swimming behaviors (Supp. Videos S8, S9). The swimming experiments of MUSAS adhering on tilapia were conducted in a 28-L Brett-type swim tunnel (Loligo Systems) filled with water at room temperature (20 °C). Water velocity was controlled using a digital DC inverter (Eurodrive; Lyman) and calibrated using a vane-wheel flow meter (Supp. Fig. S12). The working section of the swim tunnel measured 40 cm L × 20 cm W × 20 cm D. To ensure laminar, nonturbulent flow, plastic honeycomb was inserted upstream in the working section. We swam a 20-cm (body length) tilapia with the MUSAS attached to the operculum at 1.25 BL/s (25 cm/s), and 2.25 BL/s (45 cm/s) is approximately half of the critical swimming speed of tilapia, depicting their long-term fast swimming behavior at 20 °C [21].

S9. Extended *in vitro* and *in vivo* validation of MUSAS-enabled impedance sensor for gastroesophageal reflux diseases

In vitro results demonstrate that MUSAS-based impedance sensor (Fig. 5b) effectively distinguishes pH levels across acidic and alkaline environments, exhibiting high impedance sensitivity in the low-frequency range for the resistive (real) part and in the high-frequency range for the reactive (imaginary) part (Ext. Fig. 8a). We further tested the impedance pH sensor *in vivo* on a swine esophagus model. Ext. Fig. 8b depicts that the MUSAS-based impedance sensor accurately differentiates between air inhalation, water, and gastric fluid consumption, even though gastric fluid typically refluxes rapidly and discretely within a short timeframe, allowing only a limited frequency scanning range.

S10. Extended discussion on MUSAS-enabled sustained drug delivery and mRNA therapeutics

The development of MUSAS represents a highly translational effort aimed at solving critical biomedical and biological problems, especially in sustained oral drug delivery to treat chronic diseases and breaching mucosal barrier to deliver genetic therapeutics to the GI tract. The relevant applications were validated through rigorous *in vitro*, *ex vivo*, and *in vivo* studies using technically comprehensive animal models that serve as gold standards for translational research.

S10.1 Sustained release of cabotegravir for HIV/AIDS prevention

The efficacy of cabotegravir is well established in clinical studies [22]. An injectable sustained-release formulation and an oral immediate-release tablet of cabotegravir are indeed available commercially. Importantly, the focus of our work was not to study the drug's efficacy but to understand whether long-term delivery through oral application is possible. In particular, we wanted to understand if long-term delivery of cabotegravir was feasible through oral application. The primary challenge in oral long-term drug delivery is the poor retention of the drug delivery system in the gastrointestinal (GI) tract. Our work introduces a MUSAS-enabled slow-release ingestible formulation of cabotegravir, which extends its retention in the GI tract. The drug molecule absorbed into systemic circulation is cabotegravir (confirmed using LC-MS/MS analysis). As this molecule is identical to that used in the FDA-approved formulation, it is expected that the biodistribution will be identical. In other words, since the drug is absorbed as cabotegravir from both the sustained-release and immediate-release formulations, the biodistribution and efficacy will be comparable if the plasma pharmacokinetics are similar.

The *in vivo* long-term retention performance of MUSAS was validated across nine pigs, with three pigs specifically tested for drug release, demonstrating sustained delivery. Additionally, our pharmacokinetic analyses using plasma data confirm the potential to extend cabotegravir's sustained release beyond one week (Fig. 5c-I & 5c-II). Testing the efficacy of cabotegravir in pigs will require development of an HIV model in this species. Since the therapeutic efficacy of cabotegravir has already been established through clinical trials leading to FDA approval, developing an HIV pig model for further efficacy studies was considered beyond the scope of this work.

S10.2 Localized mRNA delivery in the GI tract

Achieving effective mRNA delivery to the GI tract for localized treatment presents unique challenges. To address this, we developed a device-enabled mRNA delivery system with a substantial drug-loading capacity (215 μL). *Ex vivo* study delivering fluorescent polystyrene nanoparticles to esophageal tissue suggested that MUSAS-enabled multisite microneedle delivery increased bioavailability by 5-fold compared to single-site subcutaneous injection, while the pipette-based topical application of fluorescent LNPs on the mucosa yielded tissue fluorescence comparable to the untreated control (Ext. Figs. 9a, 9b). We then confirmed that our LNPs could be frozen and thawed without loss of activity in the presence of sucrose as a cryoprotectant (Ext. Fig. 9c). Next, we applied the LNPs frozen in MUSAS in pigs, and successfully demonstrated transfection efficacy of luciferase mRNA in the GI tract confirmed by IVIS imaging and immunohistochemistry in three pigs (Fig. 5d-I, 5d-II, Ext. Figs. 9d, 9e). Notably, our work represents exceptional functional transfection (protein production) of mRNA in the GI tract, overcoming major biological barriers that have long hindered localized mRNA therapeutics for GI applications [23, 24].

It is worth noting that biodistribution studies for mRNA are most commonly conducted in small animals. This is because it requires of the firefly luciferase substate, luciferin, at a high dose (~150 mg/kg body weight) before euthanasia. As this is prohibitively expensive, we detected luciferase mRNA expression using simply local injection or tissue submersion in luciferin, which demonstrated robust efficacy. While biodistribution studies would further expand the capabilities of MUSAS, current limitations in mRNA formulation detection make such studies impractical in large animals due to ethical, financial, technological and logistical constraints. Future work of

MUSAS-enabled mRNA delivery will focus on developing mRNA formulations to enable systemic protein detection via plasma bioanalytics.

S11. Noninvasiveness and biocompatibility of MUSAS

S11.1 Noninvasiveness of MUSAS

While the microscale tissue penetration of MUSAS limits its applicability to organs with thin serosal layers and sensitive to leakage—such as vasculature or lungs—this feature presents a major advantage for breaching the mucosal barrier, enabling submucosal delivery of biological therapeutics, a key challenge in GI tract drug delivery. As shown in Figs. 4f and 4g, MUSAS can be regarded as a noninvasive microneedle platform, with a penetration depth of up to 800 μm and penetration hole diameters within 100 μm. Extensive studies on microneedles over the past decades have demonstrated their noninvasive nature and rapid tissue repair within 24 hours, with minimal adverse effects [25]. In fact, in the rare occasion where MUSAS was still present at the terminal study on the day of euthanasia and naturally detached from the pig stomach, we were able to visualize that its adhesive compartments and lamellae were covered and thoroughly lubricated with stomach contents and mucoid materials (Ext. Fig. 10b), leading to loss of its adhesion property and thus, safe passage in the GI tract. During the *in vivo* validation of MUSAS in > 58 pigs, readhesion of the devices after their detachment were never visualized during our routine X-rays checks performed every several hours in terminal studies on the day of euthanasia, or every two days to one week in survival studies (based on the animal warfare requirements to ensure animals can rest between sedation). Based on X-ray results, we estimate that the gastric emptying time for detached MUSAS is generally less than one day, which aligns with the typical gastric emptying pattern reported in pigs for safe passage of stomach contents [26]. In addition, health monitoring of our experimental pig during the adhesion and the passage of MUSAS, conducted by MIT animal committee on animal care and division of comparative medicine, were unremarkable. Pigs showed normal behaviors, food consumption and normal weight gain (Ext. Fig. 10c).

S11.2 Biocompatibility of MUSAS

The biocompatibility of MUSAS is determined by its material composition, primarily nitinol and silicone rubber (EcoFlex). In fact, both nitinol and silicone rubber have reached extensive human use in FDA-approved products, as documented in published FDA medical device material safety summaries [27, 28]. Specifically, nitinol, one of the most widely used biomaterials, has been extensively employed in endoscopy, arch wiring, cardiovascular stents, orthopedics, and artificial organs since the 1970s [29, 30]. Additionally, numerous reports over the past decade have documented the use of silicone rubber, such as FDA approved biomaterials PDMS and Eco-Flex, in implantable and ingestible electronics and biomedical devices [31, 32]. We further conducted various histological analyses with internal controls to compare the adhesion sites of MUSAS (stomach, buccal cavity, pharynx, and esophagus, localized with a tattoo, Ext. Fig. 10a) with non-adhesion areas of MUSAS, as well as downstream intestinal and colonic tissues collected after its safe passage. Histopathology of H&E-stained tissues of the gastrointestinal tract evaluated by a board-certified veterinary pathologist confirmed that MUSAS caused no evidence of significant damage including hemorrhage, inflammation or indication of tissue repair (fibrosis) (Ext. Fig. 10d–10h).

S11.3 Off-target adhesion

MUSAS has been tested in over 58 pigs and 8 fish, the optimal design achieved ⩾ 7-day GI retention in 85% of pigs, and up to 20 days in 15%—with no adverse events observed (Ext. Fig. 10). To specifically assess *in vivo f*ailure scenarios, we tested non-adhering (pre-damaged) devices, comparing to fully functional devices and confirmed that in all cases, device passed safely through the GI tract (Supp. Fig. S13). Even if partial detachment occurred in biomedical treatment applications, sustained drug delivery remained effective due to complementary devices with controlled pharmacokinetics (Fig. 5c-II). Thus, failure to adhere did not manifest in clinical risk, and adhesion consistency is validated across > 58 pigs.

S12. Failure mechanism and durability of MUSAS

S12.1 Adhesive failure

Mechanical adhesion tests (Supp. Fig. S14) and prolonged *in vitro* retention tests (Ext. Fig. 5b) demonstrate that the primary failure mechanism for adhesion under tension and shear is adhesive failure. This occurs when the vacuum-based suction is compromised due to a loss of friction necessary for a watertight seal, as well as a potential loss of intermolecular bonding initiated by alternation of setal structures at the 10–1000 nm scale. We further conducted *in vitro* studies to evaluate the fouling and adhesive failure of MUSAS incubated at body temperature (37°C) in swine gastric fluid harvested from Yorkshire pigs. Microscope and scanning electron microscope (SEM) imaging confirmed that fouling remained negligible at the micron scale for shape memory alloy (SMA) lamellae after 7 days of incubation (Supp. Fig. S15b & S15d). After 21 days, minor fouling was observed at the 50 µm scale, with minimal changes in SMA surface morphology at the 5 µm scale, indicating negligible interference with mechanical interlocking (Supp. Fig. S15d). However, surface morphology reconstruction of silicone rubber occurred over time due to the reported water permeability of Ecoflex (260 g/m^2day^{-1} [33]). Specifically, minor swelling and fouling of the silicone rubber's crease pattern appeared after 7 days and became evident after 21 days, suggesting a gradual loss of friction, which is crucial for maintaining vacuum-based mechanical adhesion in MUSAS (Supp. Fig. S15c). Additionally, changes in silicone rubber morphology were observed within the 10–1000 nm length scale (Supp. Fig. S15e). Initially, Ecoflex appeared smooth with a few detectable setal structures at high magnification (500 nm); however, fouling and surface roughening gradually developed after 7 days, and became evident after 21 days. This degradation could weaken intermolecular interactions, eventually leading to leakage and failure of the vacuum-based seal. These results suggest that the primary adhesive failure mode of MUSAS will be caused by the loss of vacuum-based mechanical adhesion due to water exchange, swelling and fouling of the silicone rubber, occurring at 500 nm to 50 µm. Notably, the material failure timeline aligns with our *in vivo* observations, where MUSAS retained stable adhesion in swine over 22 days (Fig. 4c), suggesting that critical material failure occurs only beyond the window of functional retention.

Future studies could explore improved waterproof silicone rubber and finer setal structures within the 10–1000 nm range to enhance the longevity and functionality of MUSAS. A

discussion of these findings has been added to the main manuscript and supplementary information.

S12.2 Coadhesive failure

Coadhesive failure initiated by the loss of mechanical interlocking due to lamella degradation could serve as a second failure mode, enabling controllable detachment (Fig. 4b, Ext. Fig. 5e). Furthermore, material stiffness mismatch and fatigue may contribute to coadhesive failure. To evaluate this, we conducted additional simulations to assess contact damage and durability of the lamella-flap structures upon interaction with human stomach tissue, considering the stiffness mismatch between the shape memory alloys and silicone rubber. Using Abaqus 2021 (SIMULIA), we applied a fracture mechanics model to evaluate the contact damage of tissue piercing. In accordance with previous simulations, the silicone rubber flap was modeled as EcoFlex 0030, with a density of 1.07 g/cm^3, a Young's modulus of 125 kPa, and a Poisson's ratio of 0.49 [34]. Stomach tissue properties included a density of 1.088 g/cm^3, a Young's modulus of 700 kPa, and a Poisson's ratio of 0.49 [35], and a plastic yield stress of 700 kPa [36]. The SMA lamella was assumed to remain in the martensite phase during contact, with a Young's modulus of 23 GPa, a Poisson's ratio of 0.33, and a density of 6.5 g/cm^3 [37]. We applied these to a single lamella-flap structure interacting with stomach tissue. To comprehensively evaluate contact damage, we simulated an extreme case of instant insertion where the flap-lamella structure was inserted into stomach tissue at a vertical tip speed of 2 cm/s to a depth of 1.4 mm. Notably, the silicone rubber composed of the flap is hyperelastic and can withstand strains up to 900% [38]. The simulation confirmed that stress at material interfaces with mismatched stiffness was effectively dissipated through the hyperelastic deformation of the silicone rubber, minimizing stress concentration at the lamella tip during insertion (Supp. Fig. S16a & S16b). We also conducted a fatigue analysis to assess the durability of the flap-lamella structure, under a cyclic condition of instant insertion described above. A plug-in algorithm for Nitinol in fe-safe, coupled module of Abaqus, was used to model the fatigue behavior of SMAs. Additionally, the Endurica plug-in of fe-safe was used to model the fatigue behavior of silicone rubber [39]. Fatigue analysis identified the critical area as the thinnest section of the silicone flap, where it wraps around the lamella, measuring only 100 µm in thickness. However, this area could withstand at least 86 cycles ($10^{1.94}$) before reaching 75% lifespan of its maximum durability, under extreme instant insertion contact (Supp. Fig. S16c). These results confirm that the stiffness mismatch between the lamella and soft flap has a minor impact on the durability of MUSAS, especially considering its infrequent and slow tissue contact in real-world applications.

S12.3 Prescence of Gastric Content to the Adhesion of MUSAS

While direct mucosal contact is essential for successful adhesion—a requirement shared by all GI-mucoadhesive resident systems, this does not preclude effective mucosal interaction in real-world conditions. In fact, standard clinical practice for many oral drugs already accounts for this by recommending administration in the fasted state, when gastric contents are minimal. This

includes drugs like oral semaglutide and bisphosphonates, where food interference would otherwise reduce absorption and thus efficacy [40-42].

Deploying MUSAS in a fasted state is both practical and with clinical precedent. Human gastric emptying times average ~1 hour for liquids and ~3 hours for solids, with minimal amounts of foreign matter remaining at fasted states and in between meals [43], enabling consistent windows for adhesion without food interference.

Moreover, MUSAS has been validated under physiologically relevant conditions. In multiple survival swine studies, animals were fasted prior to administration but continued normal feeding post-deployment. As shown in Fig. 4d, small amounts of gastric debris (e.g., banana shavings) were often present, yet did not impair adhesion. GI motility, including peristalsis and stomach contractions, helps position the device against suitable mucosa for attachment—even in the presence of mild debris (Fig. 4b).

In sum, while mucosal contact is essential, our data clearly demonstrate that MUSAS can achieve strategic adhesion under realistic physiological conditions, including partial food presence.

S12.4 Bioadhesive stability of MUSAS across space and time

It is worth noting that degradation of materials alone does not define adhesive failure. MUSAS's retention relies on dual mechanisms: vacuum sealing, affected by Ecoflex's integrity, and mechanical interlocking via shape memory alloy lamellae, which remained intact and morphologically stable after 21 days of incubation (Supp. Fig. S15). Importantly, *in vivo* conditions differ substantially from *in vitro* testing. In swine models, cyclical gastrointestinal contractions can help stabilize MUSAS against shear forces when oriented favorably. These same forces, when unfavorable (e.g., strong peristalsis or food impact that jeopardizes vacuum sealing or mechanical interlocking), may induce detachment. Yet even under these variable forces, MUSAS showed highly consistent retention in > 9 pigs, with 85% remaining residence beyond 7 days and 15% up to ~20 days, without affecting animal health or behavior (Fig. 4b, Ext. Fig. 5, Ext. Fig. 10), in the swine models which closely mirror human physiology, offering high translational fidelity [7]. The retention durations far exceed prior benchmarks for GI adhesives, which typically fail within 24 hours [44, 45], and are backed by pharmacokinetic validation demonstrating sustained drug release (Fig. 5c-II). In addition, regarding species and individual variability, this is an inherent feature of *in vivo* biomedical systems. Nevertheless, our extensive testing (n > 58 pigs) revealed remarkably consistent trends in adhesion performance, applicational validity, and safety, which were detailed in the main manuscript and Supplementary Information. This consistency across *in vitro*, *ex vivo* and *in vivo* studies supports the robustness and potential generalizability of the platform to human use.

Species	Museum Cat. #	Standard Length
Phtheirichthys lineatus	MCZ 33448	145 mm
	USNM 326138	332 mm
Echeneis naucrates	MCZ 30872	170 mm
	MCZ 33306	180 mm
Echeneis neucratoides	MCZ 8678	200 mm
	USNM 300452	330 mm
Remora albescens	MCZ 32104	94 mm
	MCZ 31364	60 mm
	MCZ 30798	42 mm
Remora australis	LACM 30310-18	182 mm
	CAS 26663	96 mm
	MCZ 8685	250 mm
Remora remora	MCZ 83204	133 mm
	MCZ 83212	180 mm
	MCZ 40950	149 mm
Remora brachyptera	MCZ 8668	155 mm
	USNM 202293	195 mm
	USNM 382506	245 mm
Remora osteochir	MCZ 101628	161 mm
	MCZ 43246	159 mm
	MCZ 99592	195 mm

Table S1. Information of remora specimens analyzed in micro-CT. Specimens were obtained from the Museum of Comparative Zoology (MCZ), the Natural History Museum of Los Angeles County (LACM), the United States National Museum (USNM) and California Academy of Sciences (CAS).

Feature	MUSAS (Current Study)	Wang et al. Study [46]	Wang et al. Study [47]	Su Et al. Study [48]	Li et al. Study [49]
**Primary Inspiration**	Remora attachment mechanisms, specifically focused on soft tissue interaction and use in biomedical settings	Remora attachment, designed primarily for hitchhiking applications in underwater vehicles	Subsequent study of [46], focused on developing bio-inspired robot mimicking detachment of remoras for power-reduction of motors	Subsequent study of [46], focused on understanding tissue composition of remora adhesive disk for enhanced suction	Subsequent study of [46], focused on developing aerial-aquatic robots with underwater hitchhiking capabilities
**Remora Studies**	Adhesion of alive remora (*E. nau.*) to soft tissue phantom. Studies of the whole *Echeneidae* family with evolutionary explanation for remora adhesive anatomy adaptations across different species	Adhesion of alive remora (*E. nau.*) to hard glass.	Detachment of alive remora (*E. nau.*) from adhesion to glass.	Anatomical study of tissue composition of the adhesive disk of *E. nau.*	Anatomical study of compartmental structure of the adhesive disk of *E. nau.*
**Structural Focus**	Emphasis on unfurling shape, multicompartmental adhesion, and lamella orientation of adhesive disk for adhesion to soft substrates	Focus on the erection of spinules and lamellae for enhancing frictional forces on different surfaces	N/A	One-piece adhesive disk, with composite fibers mimicking tissue composition of remora adhesive disk	Focus on multicompartmental structures for redundant adhesion
**Primary Application Focus**	Biomedical applications, particularly noninvasive gastrointestinal drug delivery and biosensing	Underwater hitchhiking and attachment for robotic platforms	Underwater hitchhiking and attachment for robotic platforms	Pneumatic soft robotics	Aerial-aquatic robots with underwater hitchhiking capabilities
**Device Size**	Miniaturized (< 25 mm Length × 9.5 mm Width)	> 10 cm in Length	> 10 cm in Length	> 10 cm in Length	> 10 cm in Length
**Lamella Design and Angle Control**	Detailed study of lamellar angles and distributions in different remora species to optimize soft substrates adhesion	Limited variation in lamella design; primary focus on achieving overall adhesion via disc structure	N/A	N/A	Limited variation in lamella design; primary focus on achieving overall adhesion via disc structure
**Soft Substrates Compatibility**	Soft, dynamic, non-intact and porous surfaces with stiffness ranging from 9 kPa to 19 MPa, average areal surface roughness S_a (3D) ranging from 2 µm to 124 µm, and maximum surface roughness S_z (3D) ranging from 30 µm to 662 µm	Tested on rough compliant surface, with stiffness of 6.5 MPa [50], and arithmetic average roughness R_a (2D) of 200 µm	N/A	N/A	N/A
**Preloading Requirements**	0.05N for adhesion establishment, 0.2N – 0.5N for full adhesion performance	N/A	N/A	1N – 30N	10N – 30N
**Actuation Mechanism**	Motor-free, leveraging endogenous force of the GI tract	Pneumatic motor	Hydraulic motor	N/A	Hydraulic motor
**Materials and Environmental Testing**	Insensitivity to pH and humidity variations, suggesting versatility in various environmental conditions for biomedical use	Tested mainly for water-based environments without sensitivity studies for biological environments	Tested mainly for water-based environments without sensitivity studies for biological environments	Tested mainly for water-based environments without sensitivity studies for biological environments	Tested mainly for water-based environments without sensitivity studies for biological environments
**Release Mechanism for Biomedical Use**	Programmable cohesive failure by leveraging biodegradable lamella materials	No detachment or clearance mechanism for biomedical applications discussed	No detachment or clearance mechanism for biomedical applications discussed	No detachment or clearance mechanism for biomedical applications discussed	No detachment or clearance mechanism for biomedical applications discussed
**In-Depth Mechanism Exploration**	Leveraging multiphysics simulation, universal mechanical test, µ-CT, particle image velocimetry (PIV), scanning electron microscope (SEM), proliometer imaging, confocal microscope for comprehensive analysis of adhesion and retention mechanisms on soft substrates, including disk shape, lamellae orientation and compartmental adhesion	Leveraging universal mechanical test for enhanced frictional interaction on rough and compliant surfaces	Leveraging PIV to mimic detachment of remora	Leveraging histology, SEM and microscope to understand tissue composition of remora adhesive disk	Leveraging frustrated total internal reflection (FTIR) and universal mechanical test to evaluate compartmental adhesion performance
**Biomedical Validation**	Comprehensive biocompatibility and safety analyses for potential biomedical applications	N/A	N/A	N/A	N/A
**Mechanical Reliability to Dynamic Interference**	*In vitro* shaking incubation test and *in vivo* endoscopic interference test	N/A	N/A	N/A	N/A
**Testing Requirement for Application Demonstration**	Unstructured *in vivo* dynamic environment including complex movements, peristalsis, variable fluid flow, and mechanical forces, using over 55 pigs and 8 fish without interfering with their normal feeding, resting, or behaviors.	Laboratory fish tank	Laboratory fish tank	N/A	Unstructured natural environment including artificial architectures, rocks in canyon and streams, and artificial plastic objects on ocean
**Retention Demonstration**	Demonstrated GI retention in pigs and body surface retention in fish (>6) under unstructured *in vivo* dynamic conditions	N/A	N/A	N/A	N/A
**Application Novelty**	Ultra-miniaturized underwater battery-free, wireless temperature biosensing, noninvasive adhesion and health-monitoring for GERD, sustained release of PrEP for HIV/AIDS, major advancement in demonstration of mRNA transfection in the gastrointestinal (GI) tract	Hitchhiking underwater vehicles	N/A	N/A	Aerial-aquatic robots with underwater hitchhiking capabilities

Table S2. Comparison of MUSAS with previous remora-inspired studies

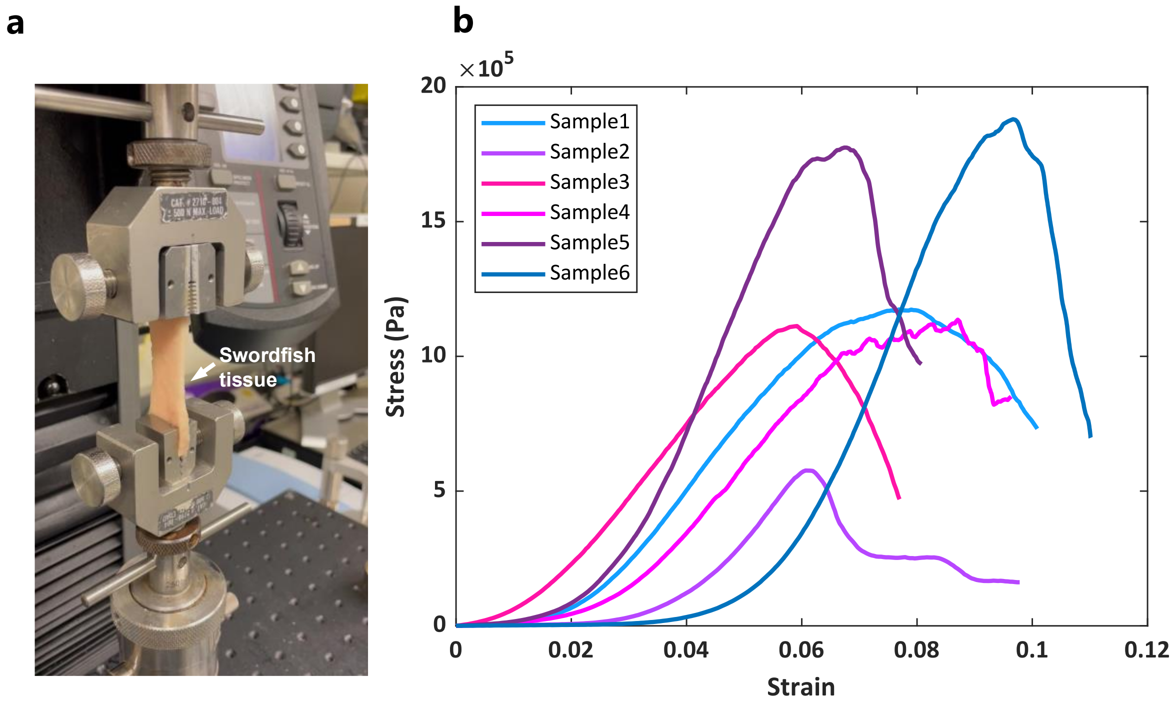

Figure S1. Measurement of tissue stiffness in swordfish (*Xiphias gladius*). a. Freshly frozen swordfish tissue (Waterfront Bistro) was thawed and cut into strips (n = 6 samples) approximately 12 × 1 × 0.15 cm in size and gripped on a 5944 Universal Testing System (Instron) for tensile testing. b. Stress-strain relationship of the sample strips.

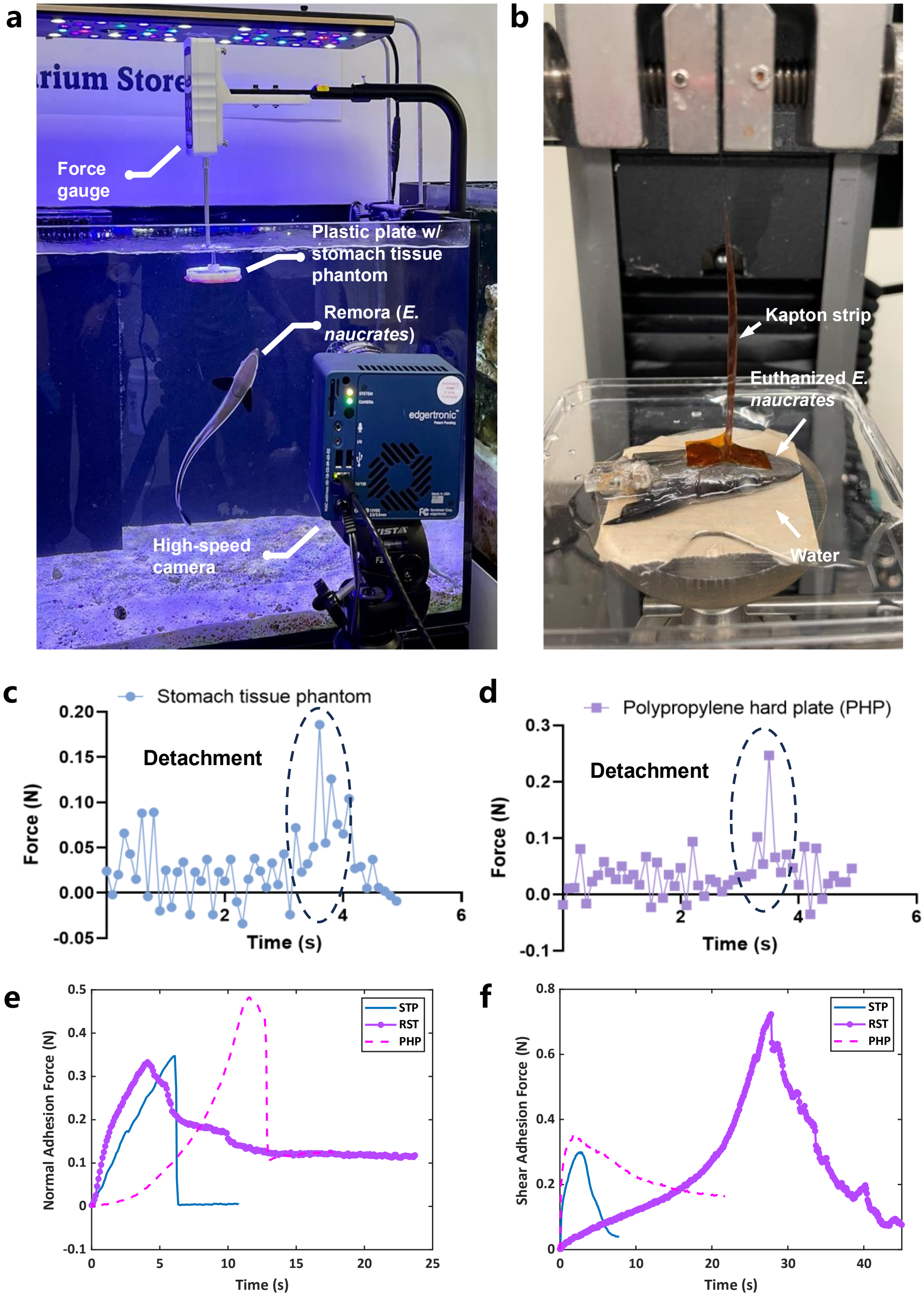

Figure S2. Characterization of live remoras (*E. Nau.*). a. Experimental setup for the characterization of live *E. Nau.* (body length ~7.6 cm), with a Series 4 force gauge (Mark-10). b. Universal mechanical testing setup (Instron) for characterizing the adhesion of euthanized *E. Nau.* on a watery plastic hard plate (PHP). c and d. Representative measurements of the normal adhesion force of live *E. Nau.* (n = 5 independent experiments). e and f. Representative measurements of normal and shear adhesion forces of euthanized *E. Nau.* on stomach tissue phantom (STP), real pig stomach tissue (RST), and a polypropylene hard plate (PHP) (n = 5 independent experiments).

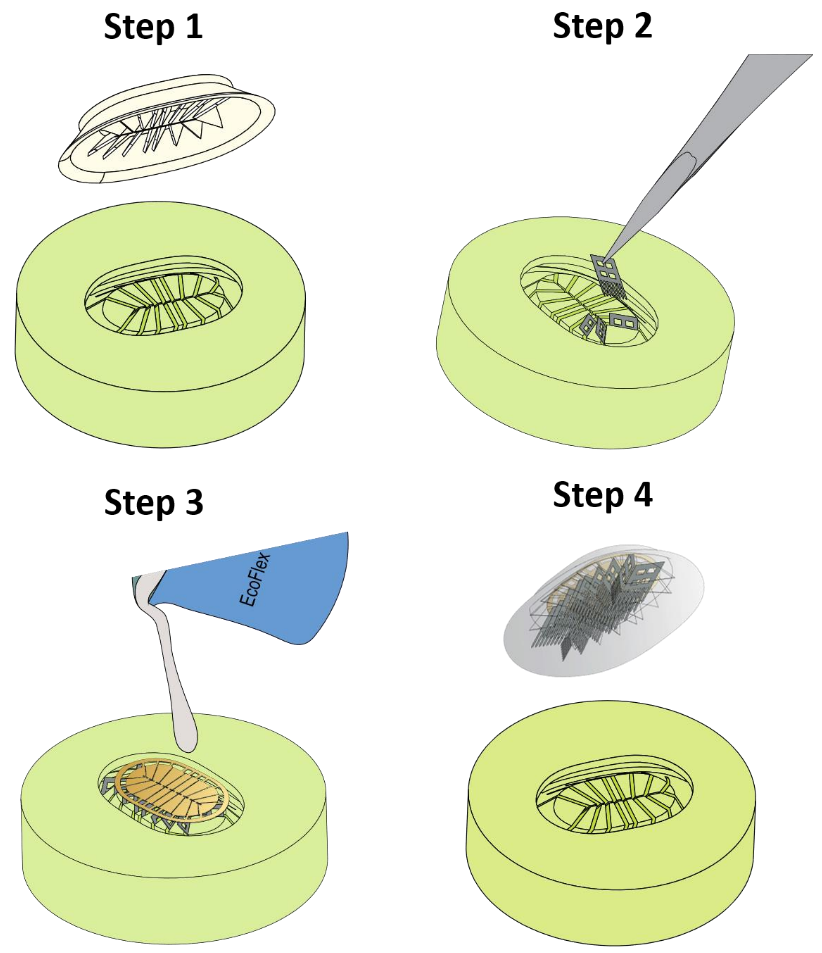

Figure S3. Fabrication of MUSAS. Step 1. Use silicone rubber (Zhermack) to fabricate a negative mold from a 3D-printed positive mold. Step 2. Insert laser-cut lamellae and the backbone structure into the negative mold. Step 3. Pour silicone rubber (Ecoflex 0030 or Zhermack Elite Double 8). Step 4. Remove and clean the cured device from the negative mold.

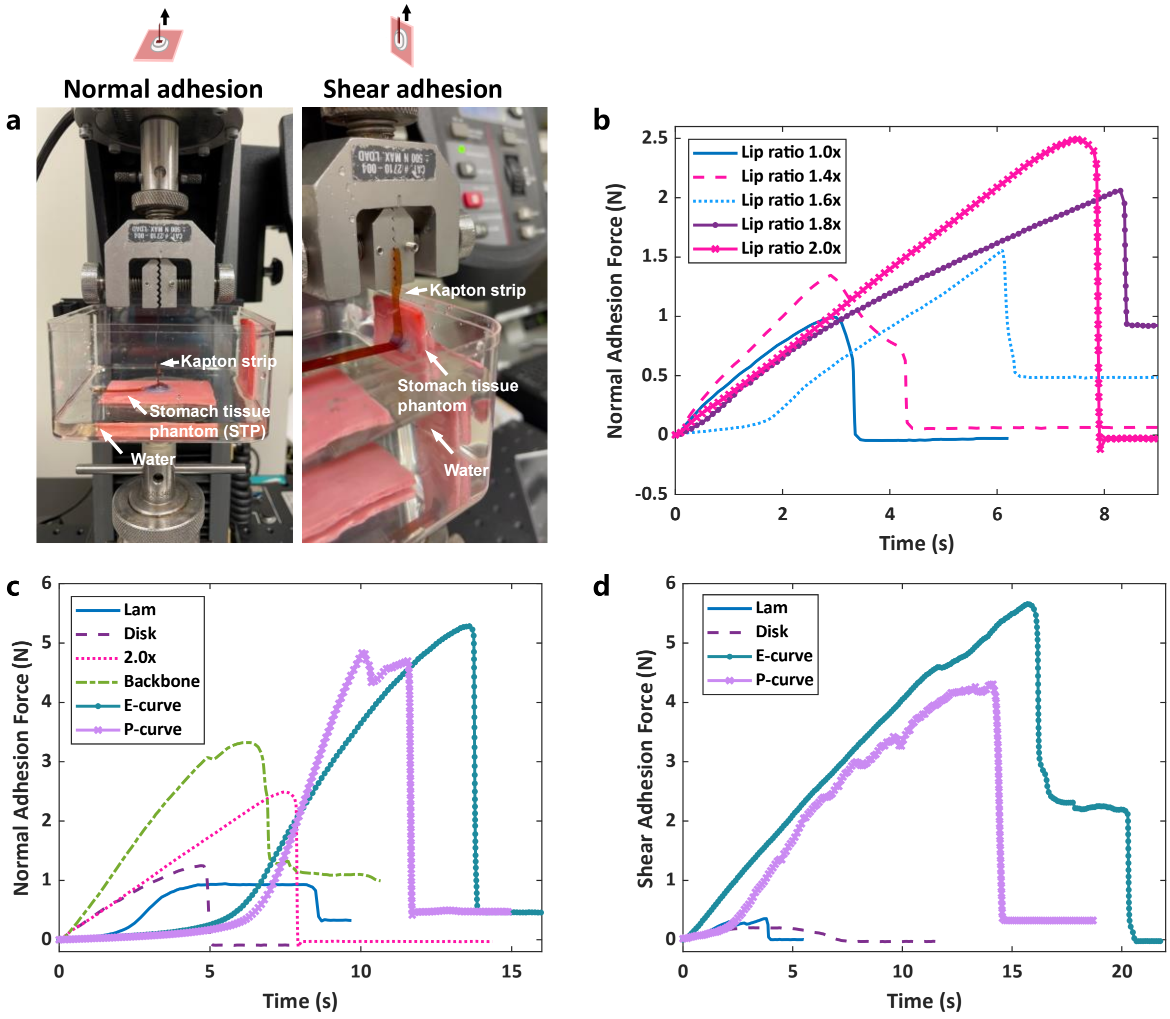

Figure S4. Adhesion performance of MUSAS equipped with different functioning components. a. Universal mechanical testing setup (Instron) for characterizing various adhesion devices. b. Representative measurements of underwater normal adhesion force to determine the optimal lip thickness ratio on STP. The relative mass of the corresponding tested device is 0.4027 g (1.0x), 0.4201 g (1.4x), 0.4158 g (1.6x), 0.4175 g (1.8x), and 0.4235 g (2.0x). The gravity used to calculate the maximum force-to-weight ratio is 9.807 m/s² (n = 5 devices per design). c and d. Representative measurements of underwater normal and shear adhesion force of various designs and components of MUSAS on stomach tissue phantom (n = 5 devices per design).

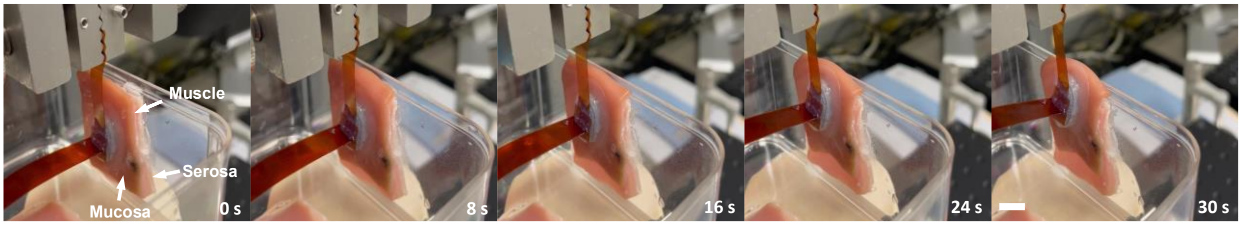

Figure S5. Dynamic morphing and layer sliding of soft substrates in maintaining adhesion. Dynamic surface morphing and tissue layer sliding between the mucosa, muscle and serosa of pig stomach tissue during MUSAS adhesion test (scale bar: 1 cm).

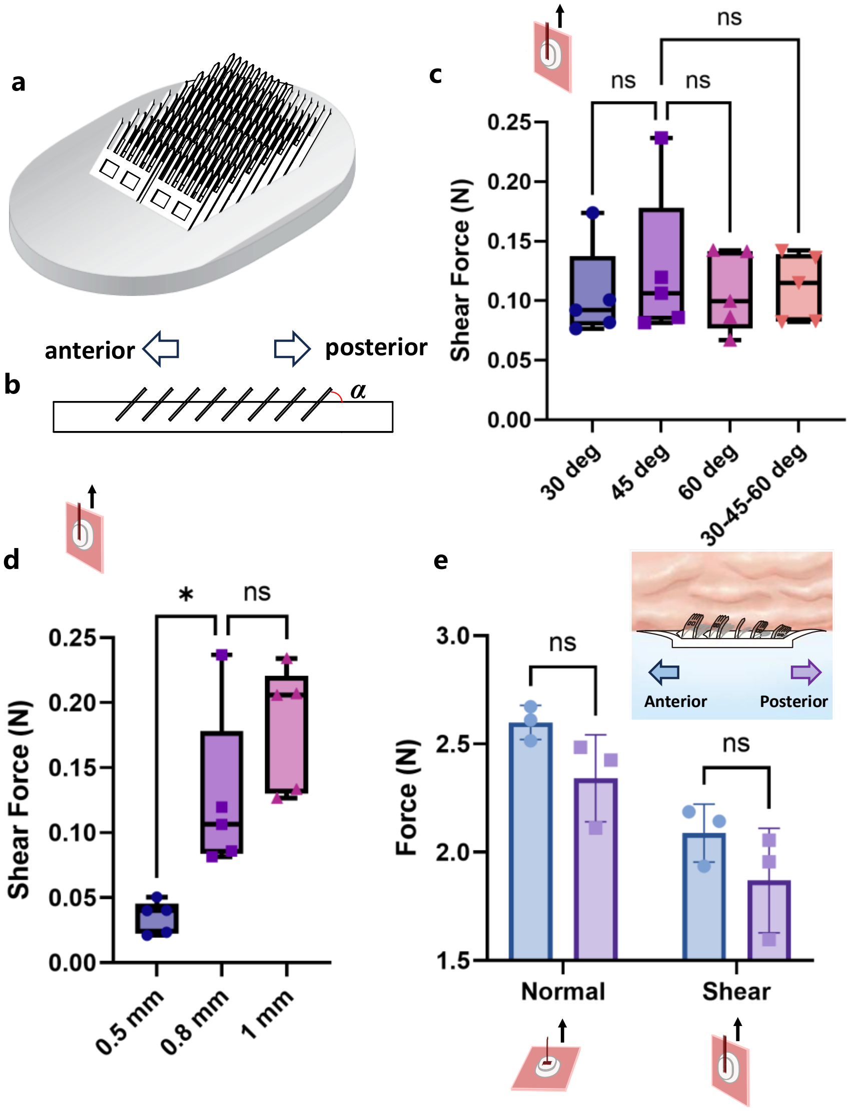

Figure S6. Shear drag performance in terms of contact angle, spinule length, and direction. a. Illustration of the lamella-only device used to characterize the impact of different lamella designs on shear performance. b. Illustration of the contact angle α of the lamella. c. No significant difference was detected in shear frictional performance relative to contact angle α (n = 5 devices per design, with 3 tested in the anterior shear drag direction and 2 tested in the posterior direction, error bars represent mean ± s.d.). d. Differences in shear frictional performance with varying spinule lengths of the lamella (n = 5 devices per design, error bars represent mean ± s.d.). e. *Ex vivo* studies of the optimal tilted-angled MUSAS (Fig. 2) showed no significant difference in adhesion direction to swine small intestine tissue (n = 3 devices, error bars represent mean ± s.d.). One-way ANOVA with Dunnett (c, d) and two-way ANOVA with Šídák multiple comparisons test (e) were used to compare different designs, statistical significance was indicated as follows: non-significant (ns) and $p \leqslant 0.05$ (*).

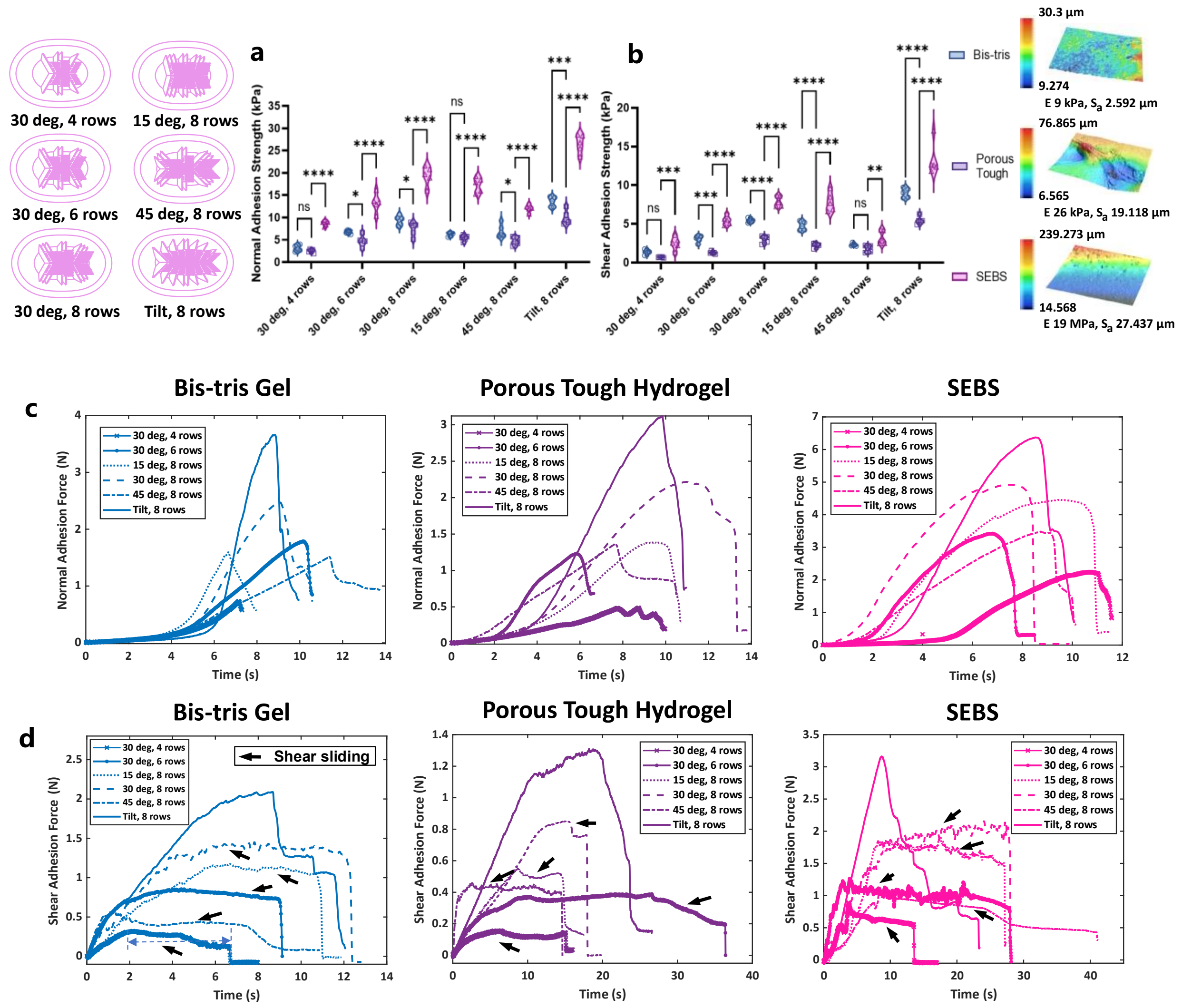

Figure S7. Mechanical characterization of adhesion performance of MUSAS with varying lamella orientation angles and row numbers, on representative soft substrates with distinct stiffness and roughness. a and b. Adhesion performance of MUSAS with varying lamella orientation angles and row configurations in normal (a) and shear (b) directions, on soft substrates of different stiffness and roughness (n = 5 devices per design, dash and dot lines represent median and Q1/Q3 quartiles). c. Representative measurements of normal adhesion forces of MUSAS with varying lamella orientations and row numbers on Bis-Tris gel, porous tough hydrogel, and SEBS (n = 5 devices per design). d. Representative measurements of shear adhesion forces of MUSAS with varying lamella orientations and row numbers on Bis-Tris gel, porous tough hydrogel, and SEBS (n = 5 devices per design). Two-way ANOVA with Dunnett multiple comparison test was used to compare adhesion across different substrates (a, b), statistical significance was indicated as follows: non-significant (ns), $p \leqslant 0.05$ (*), $p \leqslant 0.01$ (**), $p \leqslant 0.001$ (***), and $p \leqslant 0.0001$ (****).

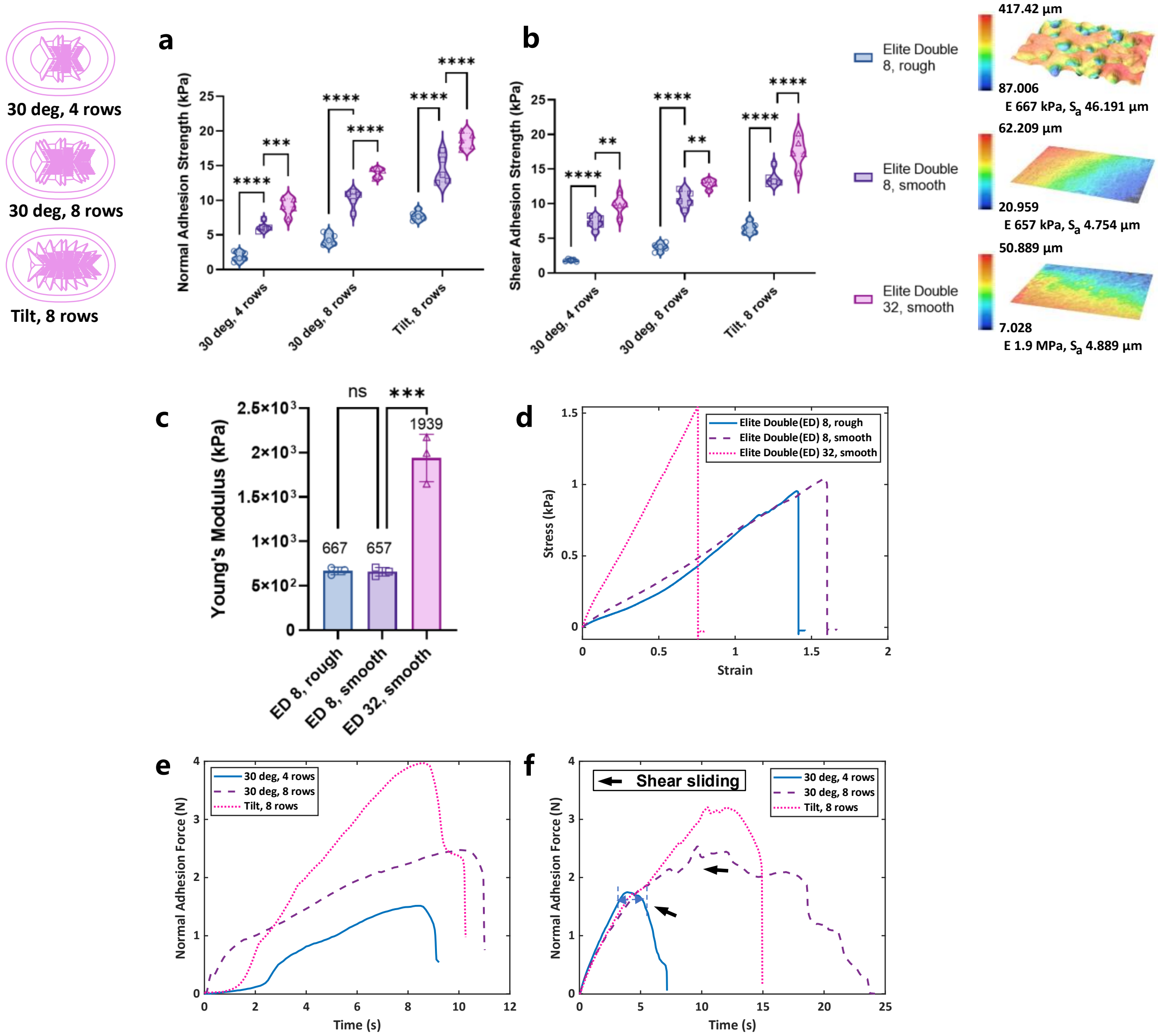

Figure S8. Extended evaluation of the adhesion performance of representative MUSAS designs on soft substrates with controlled mechanical properties: specifically, substrates with identical stiffness but distinct roughness, and substrates with identical roughness but distinct stiffness. a and b. Adhesion performance of representative MUSAS designs in normal and shear directions, on soft substrates with either identical stiffness but distinct roughness, or identical roughness but distinct stiffness (n = 5 devices per design, dash and dot lines represent median and Q1/Q3 quartiles). c. Mechanical characterization of the Young's modulus of the soft substrates used in panels a and b, with mean values labeled (n = 3 samples, error bars represent mean ± s.d.). d. Representative measurement of the Young's modulus of the substrates analyzed in panel c (n = 3 samples). e and f. Representative measurements of normal and shear adhesion forces of MUSAS on smooth Elite Double 8 silicone rubber substrates tested in panels a and b (n = 5 devices per design). Two-way (a, b) and one-way (c) ANOVA with Dunnett multiple comparison test was used to compare adhesion and stiffness across different substrates, statistical significance was indicated as follows: non-significant (ns), $p \leqslant 0.05$ (*), $p \leqslant 0.01$ (**), $p \leqslant 0.001$ (***), and $p \leqslant 0.0001$ (****).

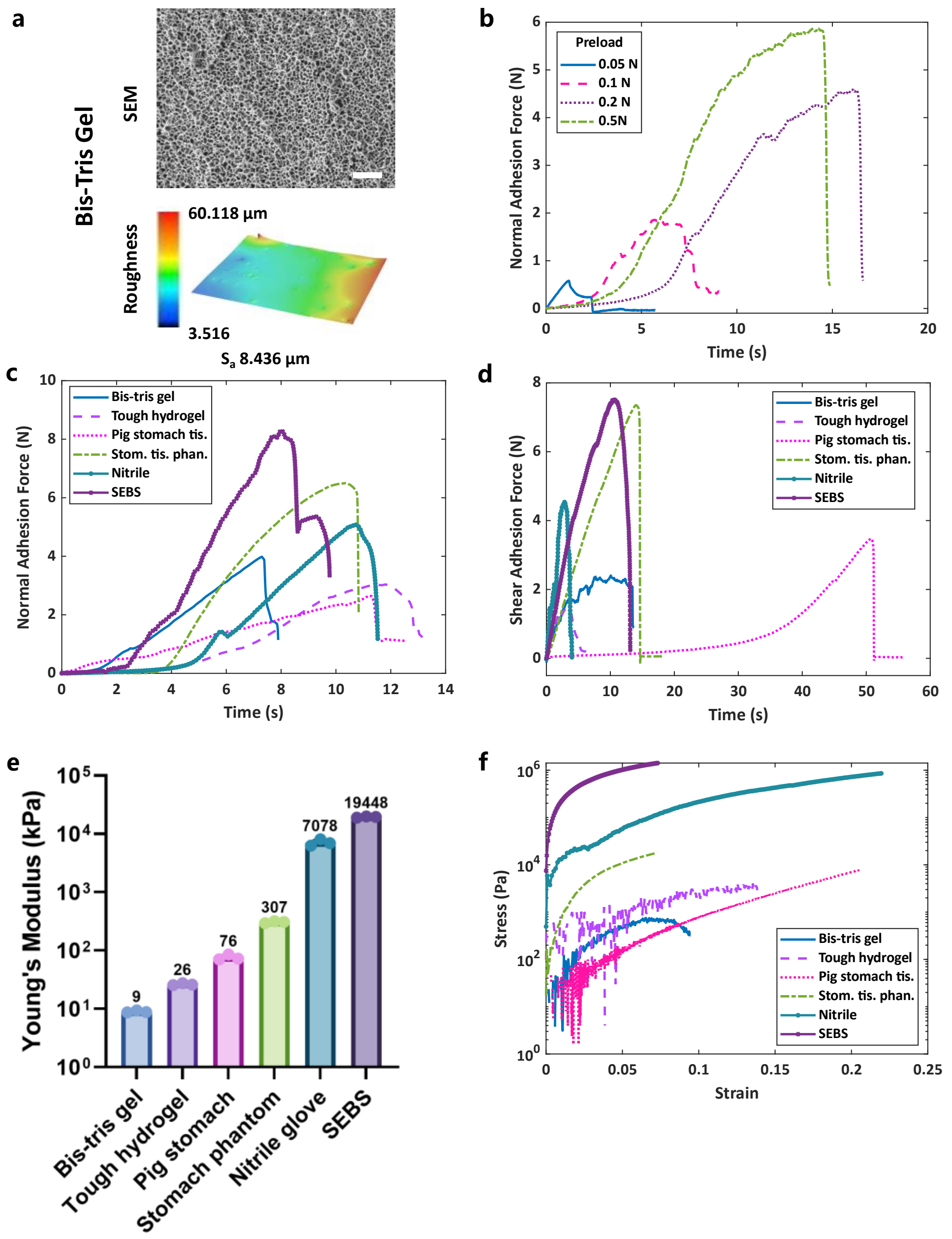

Figure S9. Adhesion performance of MUSAS on various soft substrates. a. SEM imaging and surface roughness analysis of Bis-Tris gel (scale bar: 1 µm, n = 3 samples). b. Representative measurements of normal adhesion force of MUSAS adhering to phantom stomach tissue underwater, under varying preloading conditions (n = 5 devices). c and d. Representative measurements of the normal and shear adhesion forces of MUSAS on various soft substrates (n = 5 devices). The mass of the optimal tilted-angled MUSAS used for calculating the maximum force-to-weight ratio is 0.6056 g, considering gravity of 9.807 m/s². e. Mechanical characterization of the Young's modulus of various substrates, with the mean value labeled (n = 3 samples, error bars represent mean ± s.d.). f. Representative measurement of the Young's modulus of various substrates (n = 3 samples).

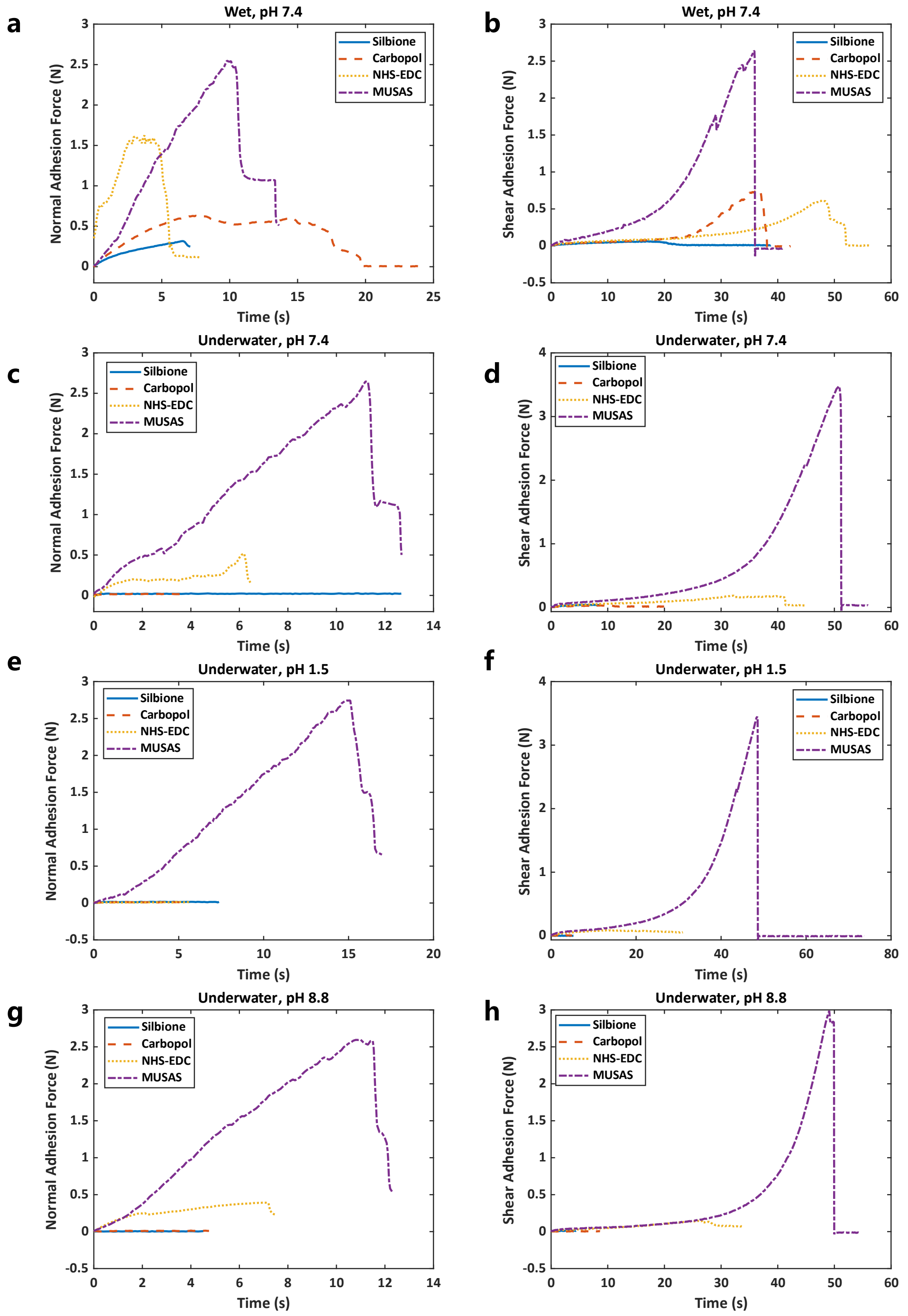

Figure S10. Adhesion performance of MUSAS under different pH and moisture conditions. a and b. Representative measurements of normal and shear adhesion forces of MUSAS, compared with medical adhesive (Silbione), hydrogen bonding (Carbopol), and covalent bonding (NHS-EDC) on swine stomach tissue (wet, pH = 7.4, n = 5 per adhesives). c and d. Representative comparison of adhesion force on swine stomach tissue (underwater, pH = 7.4, n = 5 per adhesives). e and f. Sample comparison of adhesion force on swine stomach tissue in SGF (underwater, pH = 1.5, n = 5 per adhesives); SGF was prepared with hydrochloric acid (HCl). g and h. Representative comparison of adhesion force on swine stomach tissue (underwater, pH = 8.8, n = 5 per adhesives); the alkaline solution was prepared with sodium hydroxide (NaOH).

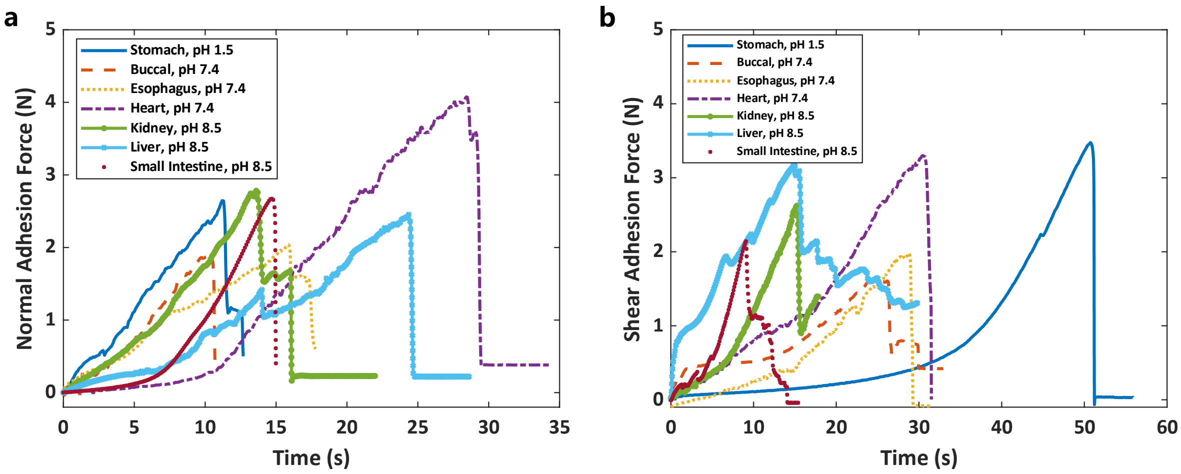

Figure S11. Underwater adhesion performance of MUSAS on different swine organs. a and b. Representative measurements of normal (a) and shear (b) adhesion forces of MUSAS on various organs (n = 5 devices per organ). The underwater pH setup for testing liver and kidney considered the pH of bile [51] and the alkaline urine of the "alkaline tide" after a meal [52].

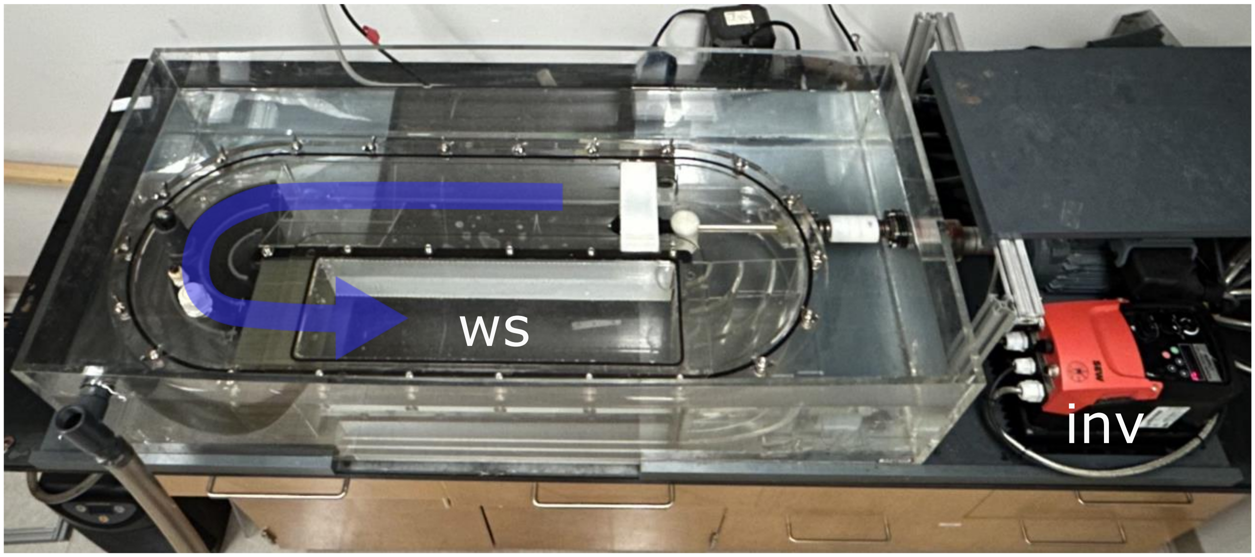

Figure S12. The 28-L Brett-type swim tunnel used for kinetic temperature readings from a MUSAS attached to the operculum of a tilapia. Water flow (blue stream) velocity was controlled by a digital DC inverter (inv). The tilapia swam in the working section (ws) at 25 cm/s (1.25 BL/s) and 45 cm/s (2.25BL/s).

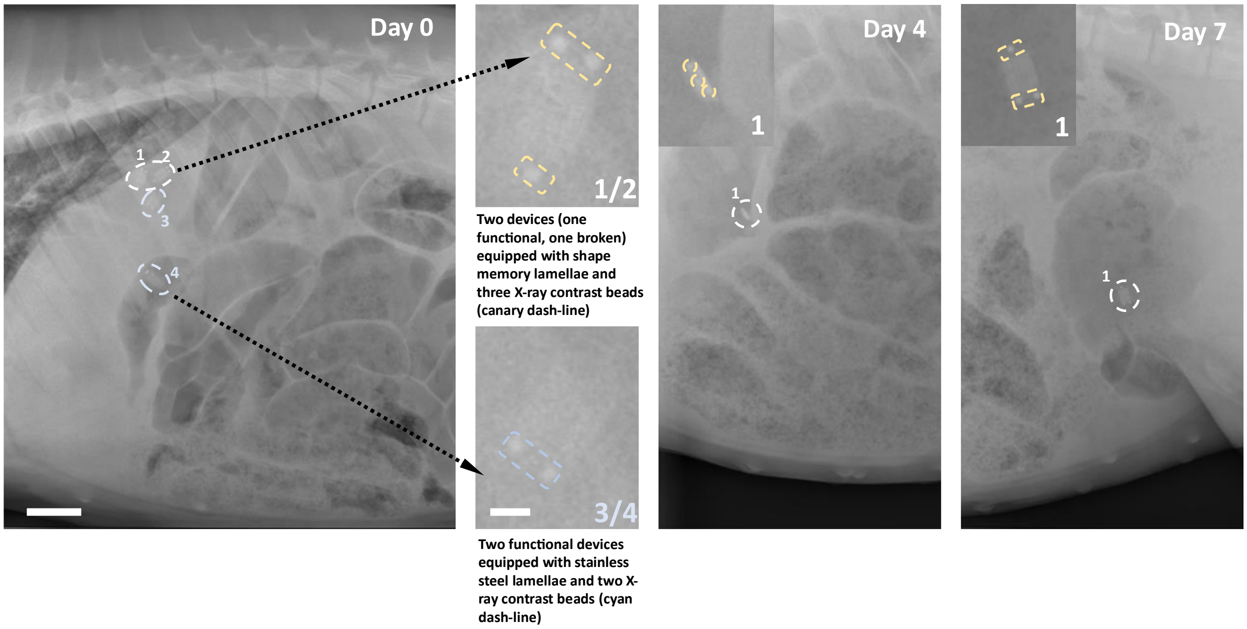

Figure S13. Retention studies in a survival swine model, comparing a broken MUSAS with a destroyed adhesive disk against fully functional devices equipped with either stainless steel or shape memory lamellae. Both the broken devices and those with stainless steel lamellae passed safely through the GI tract quickly. In contrast, the fully functional device with shape memory lamellae identified a suitable adhesion site during bowel movement and demonstrated retention for up to seven days (scale bars: left 5 cm, right 5 mm).

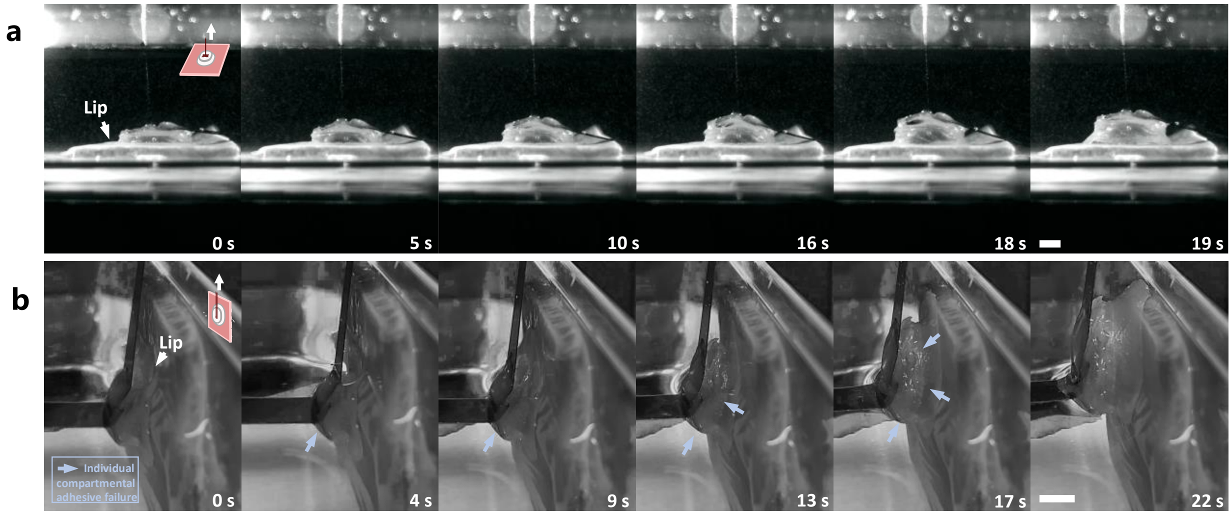

Figure S14. Adhesive failure mode of MUSAS. a and b. High-speed photograph of adhesive failure modes in normal (a) and shear (b) directions (scale bars: a, 5 mm; b, 5 mm).

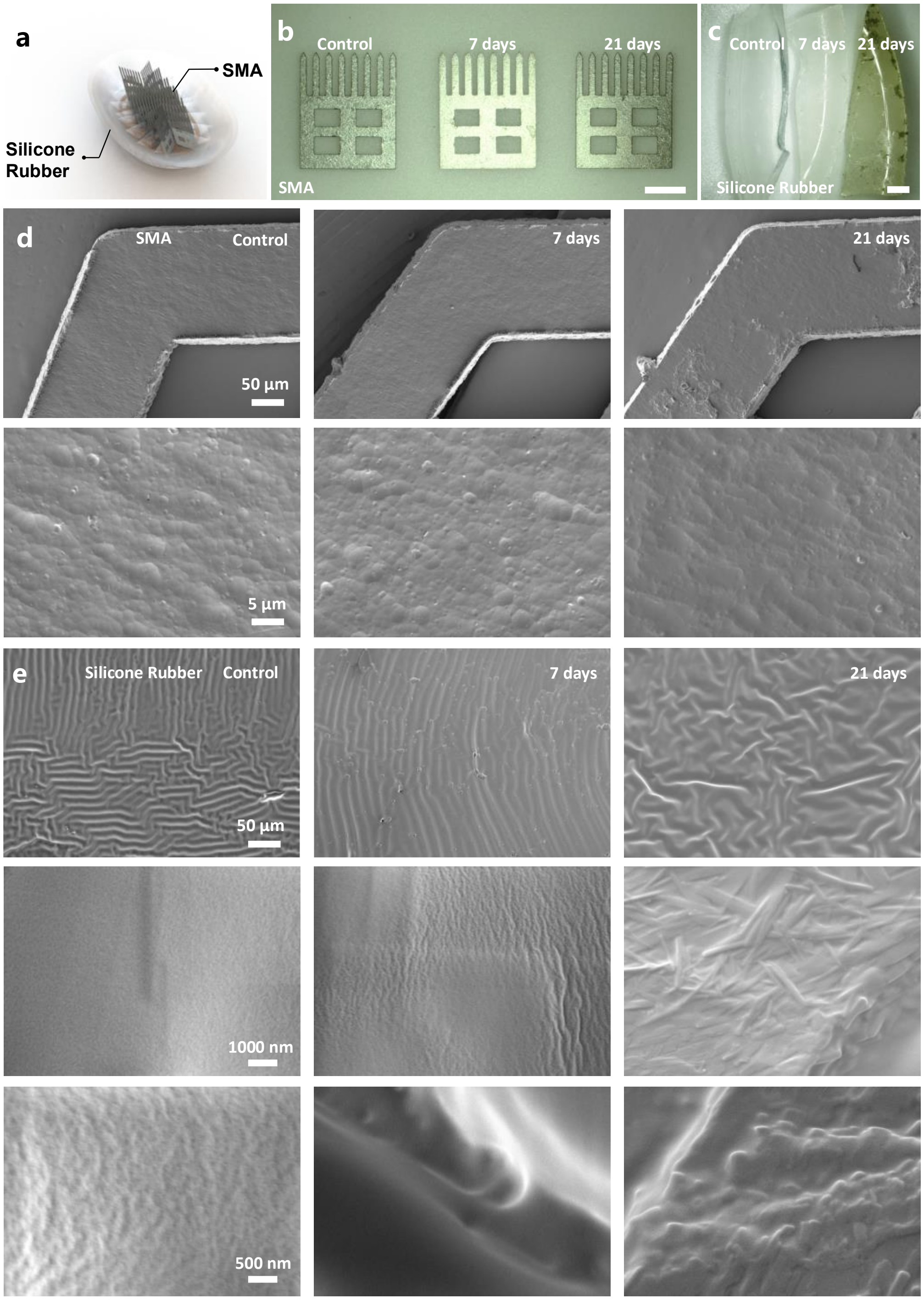

Figure S15. *In vitro* characterization of fouling of MUSAS. a. Illustration of the material components of MUSAS. b. Microscope pictures of shape memory alloy (SMA) lamellae incubated in swine gastric fluid at 37 °C for different period of time (scale bar: 1 mm, n = 2 independent samples). c. Microscope pictures of silicone rubber (Ecoflex 0030) incubated swine gastric fluid at 37 °C for different period of time (scale bar: 1 mm, n = 2 samples). d. SEM images characterizing surface morphology of SMA incubated for different period of time at micron scale, n = 2 independent samples. e. SEM images characterizing surface morphology of silicone rubber (Ecoflex 0030) incubated for different period of time at micron and nanometer scales, n = 2 samples.

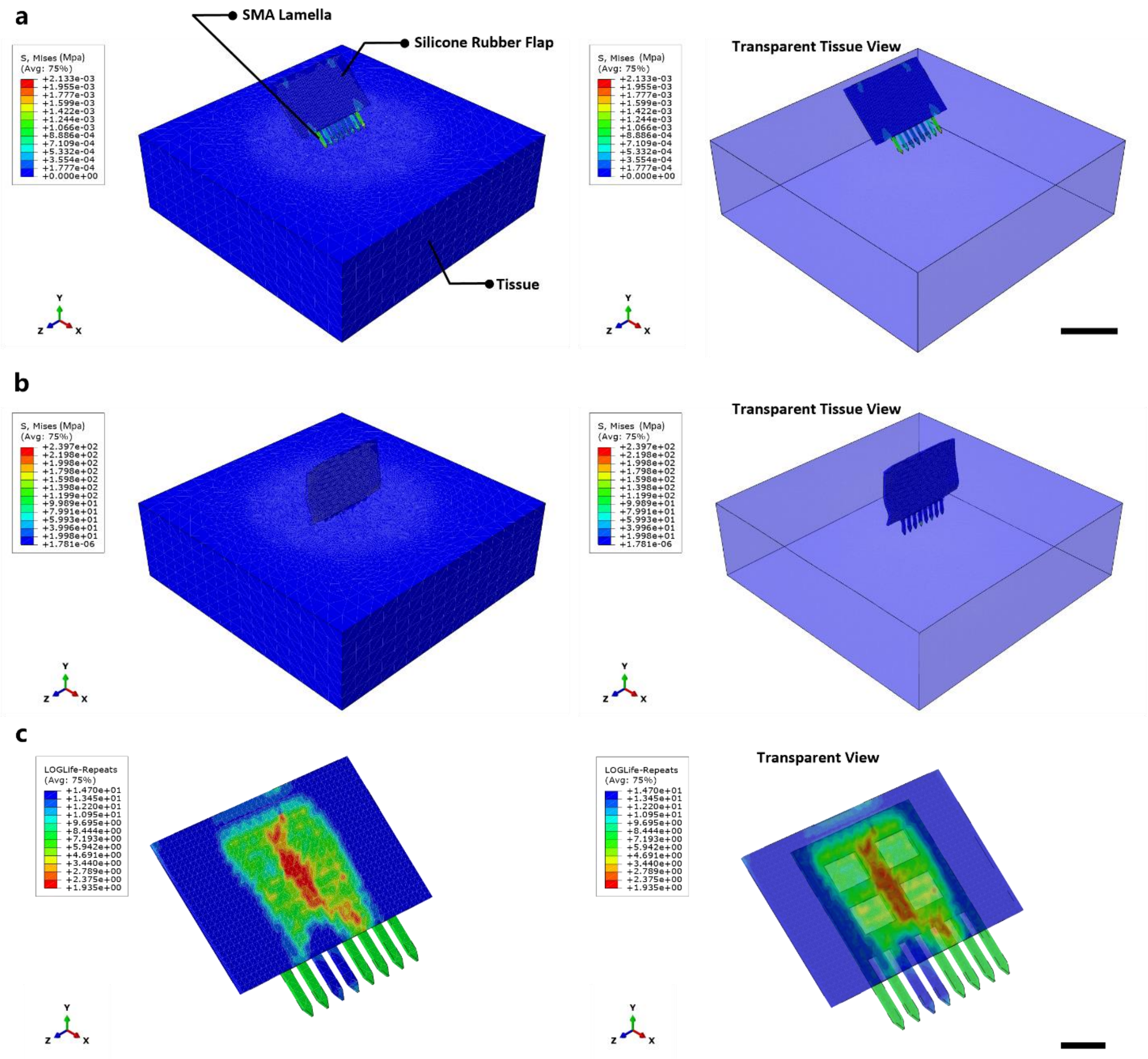

Figure S16. Contact and durability analysis of the lamella-flap structure. a. Representative deformation and stress distribution when the lamella-flap structure first contacts the stomach tissue at a tip piercing speed of -2 cm/s in the y-direction (scale bar: 3 mm). b. Representative deformation and stress distribution when the lamella-flap structure fully engages with the stomach tissue, reaching an insertion depth of 1.4 mm at a tip piercing speed of -2 cm/s in the y-direction. c. Fatigue analysis assessing the durability of the lamella-flap structure, demonstrating an average lifespan of 75% of its log10 cycle repeats under a cyclic contact procedure transitioning from a to b (scale bar: 1 mm).

Supplementary Videos

Video S1. Characterization of differential adhesion of live remoras to soft and hard substrates.

Video S2. Finite element analysis of underwater adhesion on stomach tissue, comparing a furled adhesive disk with compartments, an unfurled adhesive disk with compartments, and a benchmark one-piece adhesive disk without compartments.

Video S3. Actuation of temperature-responsive nitinol lamellae.

Video S4. Shear sliding of parallel-angled MUSAS design.

Video S5. Underwater and wet adhesion of MUSAS on various soft substrates.

Video S6. MUSAS resistance to dynamic interference.

Video S7. *In vivo* adhesion of MUSAS to different parts of the swine gastrointestinal tract.

Video S8. Adhesion of MUSAS to different parts of a tilapia body.

Video S9. Underwater MUSAS-enabled kinetic temperature sensing, and swimming tests with MUSAS-adhered tilapia.

Supplementary References

[1] Souli, M.H. and Benson, D.J. eds., 2013. Arbitrary Lagrangian Eulerian and fluid-structure interaction: numerical simulation. John Wiley & Sons.

[2] https://www.factor2.com/v/vspfiles/sds%20tds%202023/tds%202023/RTV-4717%20ELKEM.pdf

[3] Li, J., Celiz, A.D., Yang, J., Yang, Q., Wamala, I., Whyte, W., Seo, B.R., Vasilyev, N.V., Vlassak, J.J., Suo, Z. and Mooney, D.J., 2017. Tough adhesives for diverse wet surfaces. Science, 357(6349), pp.378-381.

[4] Yuk, H., Varela, C.E., Nabzdyk, C.S., Mao, X., Padera, R.F., Roche, E.T. and Zhao, X., 2019. Dry double-sided tape for adhesion of wet tissues and devices. Nature, 575(7781), pp.169-174.

[5] Friis, S.J., Hansen, T.S., Poulsen, M., Gregersen, H., Brüel, A. and Nygaard, J.V., 2023. Biomechanical properties of the stomach: A comprehensive comparative analysis of human and porcine gastric tissue. Journal of the Mechanical Behavior of Biomedical Materials, 138, p.105614.

[6] Thielicke, William, and René Sonntag. "Particle Image Velocimetry for MATLAB: Accuracy and enhanced algorithms in PIVlab." (2021).

[7] Lunney, J.K., Van Goor, A., Walker, K.E., Hailstock, T., Franklin, J. and Dai, C., 2021. Importance of the pig as a human biomedical model. Science translational medicine, 13(621), p.eabd5758.

[8] Stillhart, Cordula, et al. "Impact of gastrointestinal physiology on drug absorption in special populations—An UNGAP review." European Journal of Pharmaceutical Sciences 147 (2020): 105280.

[9] Abuhelwa, Ahmad Y., et al. "Food, gastrointestinal pH, and models of oral drug absorption." European journal of pharmaceutics and biopharmaceutics 112 (2017): 234-248.

[10] Patra, C.N., Priya, R., Swain, S., Jena, G.K., Panigrahi, K.C. and Ghose, D., 2017. Pharmaceutical significance of Eudragit: A review. Future Journal of Pharmaceutical Sciences, 3(1), pp.33-45.

[11] Abramson, A., Caffarel-Salvador, E., Soares, V., Minahan, D., Tian, R.Y., Lu, X., Dellal, D., Gao, Y., Kim, S., Wainer, J. and Collins, J., 2019. A luminal unfolding microneedle injector for oral delivery of macromolecules. Nature medicine, 25(10), pp.1512-1518.

[12] Thakral, S., Thakral, N.K. and Majumdar, D.K., 2013. Eudragit®: a technology evaluation. Expert opinion on drug delivery, 10(1), pp.131-149.

[13] Gecse, K.B. and Vermeire, S., 2018. Differential diagnosis of inflammatory bowel disease: imitations and complications. The lancet Gastroenterology & hepatology, 3(9), pp.644-653.

[14] Petricevic, B., Kabiljo, J., Zirnbauer, R., Walczak, H., Laengle, J. and Bergmann, M., 2022, November. Neoadjuvant immunotherapy in gastrointestinal cancers–The new standard of care?. In Seminars in cancer biology (Vol. 86, pp. 834-850). Academic Press.

[15] Necula, L., Matei, L., Dragu, D., Neagu, A.I., Mambet, C., Nedeianu, S., Bleotu, C., Diaconu, C.C. and Chivu-Economescu, M., 2019. Recent advances in gastric cancer early diagnosis. World journal of gastroenterology, 25(17), p.2029.

[16] Layke, J.C. and Lopez, P.P., 2004. Gastric cancer: diagnosis and treatment options. American family physician, 69(5), pp.1133-1141.

[17] Sitti, M. and Wiersma, D.S., 2020. Pros and cons: Magnetic versus optical microrobots. Advanced Materials, 32(20), p.1906766.

[18] https://www.utsouthwestern.edu/ctplus/stories/2021/7t-mri.html#:~:text=Only%20about%2030%20institutions%20in,than%20a%20millimeter%20of%20tissue.
[19] Bosch de Basea Gomez, M., Thierry-Chef, I., Harbron, R., Hauptmann, M., Byrnes, G., Bernier, M.O., Le Cornet, L., Dabin, J., Ferro, G., Istad, T.S. and Jahnen, A., 2023. Risk of hematological malignancies from CT radiation exposure in children, adolescents and young adults. Nature Medicine, 29(12), pp.3111-3119.
[20] Jarow, J.P. and Baxley, J.H., 2015, March. Medical devices: US medical device regulation. In Urologic Oncology: Seminars and Original Investigations (Vol. 33, No. 3, pp. 128-132). Elsevier.
[21] Alsop, Derek H., James D. Kieffer, and Chris M. Wood. "The effects of temperature and swimming speed on instantaneous fuel use and nitrogenous waste excretion of the Nile tilapia." Physiological and Biochemical Zoology 72.4 (1999): 474-483.
[22] Taki, E., Soleimani, F., Asadi, A., Ghahramanpour, H., Namvar, A. and Heidary, M., 2022. Cabotegravir/Rilpivirine: the last FDA-approved drug to treat HIV. Expert Review of Anti-infective Therapy, 20(8), pp.1135-1147.
[23] Abramson, Alex, et al. "Oral mRNA delivery using capsule-mediated gastrointestinal tissue injections." Matter 5.3 (2022): 975-987.
[24] Schultz, D., Kempen, P. J., Primdahl, S., Pereverzina, M., Uhrenfeldt, A. H., Alba, E. M., ... & Urquhart, A. J. (2024). Gastrointestinal device-mediated delivery of mRNA-lipid nanoparticles achieves distinct expression and biodistribution in mice and pigs. ACS Applied Materials & Interfaces, 16(49), 67192-67202.
[25] Kim, Yeu-Chun, Jung-Hwan Park, and Mark R. Prausnitz. "Microneedles for drug and vaccine delivery." Advanced drug delivery reviews 64.14 (2012): 1547-1568.
[26] Gregory, P.C., McFadyen, M. and Rayner, D.V., 1990. Pattern of gastric emptying in the pig: relation to feeding. British Journal of Nutrition, 64(1), pp.45-58.
[27] https://www.fda.gov/media/158490/download?attachment
[28] https://www.fda.gov/media/152353/download?attachment
[29] Ryhänen, J. "Biocompatibility of nitinol." Minimally Invasive Therapy & Allied Technologies 9.2 (2000): 99-105.
[30] Duerig, T., Pelton, A. and Stöckel, D.J.M.S., 1999. An overview of nitinol medical applications. Materials Science and Engineering: A, 273, pp.149-160.
[31] Dagdeviren, Canan, et al. "Conformal piezoelectric systems for clinical and experimental characterization of soft tissue biomechanics." Nature materials 14.7 (2015): 728-736.
[32] Zhang, Yamin, et al. "Advances in bioresorbable materials and electronics." Chemical Reviews 123.19 (2023): 11722-11773.
[33] Shao, Y., Yan, S., Li, J., Silva-Pedraza, Z., Zhou, T., Hsieh, M., Liu, B., Li, T., Gu, L., Zhao, Y. and Dong, Y., 2022. Stretchable encapsulation materials with high dynamic water resistivity and tissue-matching elasticity. ACS applied materials & interfaces, 14(16), pp.18935-18943.
[34] Heikenfeld, J., Jajack, A., Rogers, J., Gutruf, P., Tian, L., Pan, T., Li, R., Khine, M., Kim, J. and Wang, J., 2018. Wearable sensors: modalities, challenges, and prospects. Lab on a Chip, 18(2), pp.217-248.

[35] Guimarães, C.F., Gasperini, L., Marques, A.P. and Reis, R.L., 2020. The stiffness of living tissues and its implications for tissue engineering. Nature Reviews Materials, 5(5), pp.351-370.
[36] Egorov, V.I., Schastlivtsev, I.V., Prut, E.V., Baranov, A.O. and Turusov, R.A., 2002. Mechanical properties of the human gastrointestinal tract. Journal of biomechanics, 35(10), pp.1417-1425.
[37] Lagoudas, Dimitris C. "Shape memory alloys." Science and Business Media, LLC (2008).
[38] https://www.smooth-on.com/products/ecoflex-00-30/
[39] https://endurica.com/wp-content/uploads/2023/11/Silicone-Application-Spotlight-2023-10.pdf
[40] Brunton, S.A., Mosenzon, O. and Wright Jr, E.E., 2020. Integrating oral semaglutide into clinical practice in primary care: for whom, when, and how?. Postgraduate Medicine, 132(sup2), pp.48-60.
[41] Grannell, L., 2019. When should I take my medicines?. Australian prescriber, 42(3), p.86.
[42] Ismail, M.Y.M. and Yaheya, M., 2009. Drug-food interactions and role of pharmacist. Asian J Pharm Clin Res, 2(4), pp.1-10.
[43] Goyal, R.K., Guo, Y. and Mashimo, H., 2019. Advances in the physiology of gastric emptying. Neurogastroenterology & Motility, 31(4), p.e13546.
[44] Nan, K., Feig, V.R., Ying, B., Howarth, J.G., Kang, Z., Yang, Y. and Traverso, G., 2022. Mucosa-interfacing electronics. Nature Reviews Materials, 7(11), pp.908-925.
[45] Patil, H., Tiwari, R.V. and Repka, M.A., 2016. Recent advancements in mucoadhesive floating drug delivery systems: A mini-review. Journal of Drug Delivery Science and Technology, 31, pp.65-71.
[46] Wang, Y., Yang, X., Chen, Y., Wainwright, D. K., Kenaley, C. P., Gong, Z., ... & Wen, L. (2017). A biorobotic adhesive disc for underwater hitchhiking inspired by the remora suckerfish. Science Robotics, 2(10), eaan8072.
[47] Wang, S., Li, L., Sun, W., Wainwright, D., Wang, H., Zhao, W., Chen, B., Chen, Y. and Wen, L., 2020. Detachment of the remora suckerfish disc: kinematics and a bio-inspired robotic model. Bioinspiration & Biomimetics, 15(5), p.056018.
[48] Su, S., Wang, S., Li, L., Xie, Z., Hao, F., Xu, J., Wang, S., Guan, J. and Wen, L., 2020. Vertical fibrous morphology and structure-function relationship in natural and biomimetic suction-based adhesion discs. Matter, 2(5), pp.1207-1221.
[49] Li, L., Wang, S., Zhang, Y., Song, S., Wang, C., Tan, S., ... & Wen, L. (2022). Aerial-aquatic robots capable of crossing the air-water boundary and hitchhiking on surfaces. Science robotics, 7(66), eabm6695.
[50] Elastosil M4601https://www.wacker.com/h/en-us/silicone-rubber/room-temperature-curing-silicone-rubber-rtv-2/elastosil-m-4601-ab/p/000018458
[51] Blanco, Antonio, and Gustavo Blanco. Medical biochemistry. Academic Press, 2017.
[52] Goldman, Lee, and Andrew I. Schafer. Goldman's cecil medicine E-book. Elsevier Health Sciences, 2011.
