# Supplementary material for: Mechanical underwater adhesive devices for soft substrates
Source: Nature. 2025 Jul 23;643(8074):1271–80. doi: 10.1038/s41586-025-09304-4 (PMC12310550; doi:10.1038/s41586-025-09304-4)
Supplement: Supplementary file 2 — Reporting Summary [file 41586_2025_9304_MOESM2_ESM.pdf]

nature portfolio

Corresponding author(s): Giovanni Traverso

Last updated by author(s): May 26, 2025

Reporting Summary

Nature Portfolio wishes to improve the reproducibility of the work that we publish. This form provides structure for consistency and transparency in reporting. For further information on Nature Portfolio policies, see our Editorial Policies and the Editorial Policy Checklist.

Statistics

For all statistical analyses, confirm that the following items are present in the figure legend, table legend, main text, or Methods section.

n/a	Confirmed	
☐	☒	The exact sample size (n) for each experimental group/condition, given as a discrete number and unit of measurement
☐	☒	A statement on whether measurements were taken from distinct samples or whether the same sample was measured repeatedly
☐	☒	The statistical test(s) used AND whether they are one- or two-sided<br>*Only common tests should be described solely by name; describe more complex techniques in the Methods section.*
☐	☒	A description of all covariates tested
☐	☒	A description of any assumptions or corrections, such as tests of normality and adjustment for multiple comparisons
☐	☒	A full description of the statistical parameters including central tendency (e.g. means) or other basic estimates (e.g. regression coefficient) AND variation (e.g. standard deviation) or associated estimates of uncertainty (e.g. confidence intervals)
☐	☒	For null hypothesis testing, the test statistic (e.g. F, t, r) with confidence intervals, effect sizes, degrees of freedom and P value noted<br>*Give P values as exact values whenever suitable.*
☒	☐	For Bayesian analysis, information on the choice of priors and Markov chain Monte Carlo settings
☒	☐	For hierarchical and complex designs, identification of the appropriate level for tests and full reporting of outcomes
☒	☐	Estimates of effect sizes (e.g. Cohen's d, Pearson's r), indicating how they were calculated

Our web collection on statistics for biologists contains articles on many of the points above.

Software and code

Policy information about availability of computer code

Data collection	Bluehill V3.11 (Instron); MESUR Lite 2.0.0 (Mark-10); TSL ASCII Software Development Kit – for Android v2.8.0 (Technology Solutions UK LTD); Sciospec software 2.0.8 (Sciospec); SkyScan 1173 and SkyScan 1276 application software (Bruker); VK Viwer 2.2.0.135 (Keyence); LivingImage 4.8.2 (PerkinElmer); Abaqus 2021 (SIMULIA); COMSOL multiphysics 6.2 (COMSOL); CST studio suite 2022 (SIMULIA)
Data analysis	MATLAB R2022a (Mathworks), Prism 9.3 (Graphpad), ImageJ 1.54

For manuscripts utilizing custom algorithms or software that are central to the research but not yet described in published literature, software must be made available to editors and reviewers. We strongly encourage code deposition in a community repository (e.g. GitHub). See the Nature Portfolio guidelines for submitting code & software for further information.

Data

Policy information about availability of data

All manuscripts must include a data availability statement. This statement should provide the following information, where applicable:

- Accession codes, unique identifiers, or web links for publicly available datasets
- A description of any restrictions on data availability
- For clinical datasets or third party data, please ensure that the statement adheres to our policy

All data supporting the findings of this study are available within this paper, the Extended Data Figures and Supplementary Information. Source data underlying the graphical representations used in the figures are available in https://github.com/TroyKang/MUSAS.

Research involving human participants, their data, or biological material

Policy information about studies with human participants or human data. See also policy information about sex, gender (identity/presentation), and sexual orientation and race, ethnicity and racism.

Reporting on sex and gender	N/A
Reporting on race, ethnicity, or other socially relevant groupings	N/A
Population characteristics	N/A
Recruitment	N/A
Ethics oversight	N/A

Note that full information on the approval of the study protocol must also be provided in the manuscript.

Field-specific reporting

Please select the one below that is the best fit for your research. If you are not sure, read the appropriate sections before making your selection.

☒ Life sciences ☐ Behavioural & social sciences ☐ Ecological, evolutionary & environmental sciences

For a reference copy of the document with all sections, see nature.com/documents/nr-reporting-summary-flat.pdf

Life sciences study design

All studies must disclose on these points even when the disclosure is negative.

Sample size	Sample sizes were not determined due to the proof-of-concept nature of this study. Here, we are conducting small-scale experiments and exploratory investigations to assess the feasibility and viability of the device concept. The primary objective is to gather initial evidence or insights, rather than to determine a sample size based on statistical considerations.
Data exclusions	No data were excluded.
Replication	Ex vivo studies for mechanical characterization were performed on freshly harvested tissue (< 1-hour post-euthanasia) without surface washout, tissue trimming, or liquid removal. n = 2-4 tissue samples were used per MUSAS design or adhesives of interest.<br>In vivo studies of MUSAS were performed in over 58 pigs at various GI locations (buccal cavity, esophagus, stomach, and small intestine) and 8 fish, with GI retention evaluated in over 9 pigs and body surface retention in over 6 fish in total under survival conditions—defined as uninterrupted normal feeding, resting, and behaviors. In addition, treatment studies of MUSAS were validated in n = 3 pigs.<br>Specific n values and appropriate statistics (mean, median, quartiles, whiskers, error bars) with detail methods are described in the figures legends. All attempts at replication were successful and all experimental data were reproducible between independent experiments.
Randomization	Randomization of devices and animals was conducted to minimize physiological bias and enhance the validity of experimental results. Additionally, controlled experimental conditions (e.g., animal pre-treatment) and counterbalancing (weight, tissue harvesting order) were introduced where deemed meaningful. (See Methods section)
Blinding	No blinding was conducted as the studies focused on objective measurements and observations where inanimate objects were studied.

Reporting for specific materials, systems and methods

We require information from authors about some types of materials, experimental systems and methods used in many studies. Here, indicate whether each material, system or method listed is relevant to your study. If you are not sure if a list item applies to your research, read the appropriate section before selecting a response.

Materials & experimental systems

n/a	Involved in the study	
☐	☒	Antibodies
☐	☒	Eukaryotic cell lines
☒	☐	Palaeontology and archaeology
☐	☒	Animals and other organisms
☒	☐	Clinical data
☒	☐	Dual use research of concern
☒	☐	Plants

Methods

n/a	Involved in the study	
☒	☐	ChIP-seq
☒	☐	Flow cytometry
☒	☐	MRI-based neuroimaging

Antibodies

Antibodies used	Firefly luciferase polyclonal primary antibody (Thermal Fisher Scientific) with 1:2000 dilution ratio, conjugated with goat anti-rabbit IgG (H+L) cross-absorbed, Alexa Fluor™ 647 secondary antibody (Thermo Fisher Scientific) with 1:500 dilution were used for validating luciferase transfection in pig tissue
Validation	This Antibody is commercially available and was verified by Cell treatment to ensure that the antibody binds to the antigen stated by the manufacture. Validation statements and relevant citations published on the manufacturer's website are listed as follows.<br>Firefly luciferase polyclonal primary antibody: https://www.thermofisher.com/antibody/product/Firefly-luciferase-Antibody-Polyclonal/PA5-32209<br>Alexa Fluor™ 647 secondary antibody: https://www.thermofisher.com/antibody/product/Goat-anti-Rabbit-IgG-H-L-Cross-Adsorbed-Secondary-Antibody-Polyclonal/A-21244

Eukaryotic cell lines

Policy information about cell lines and Sex and Gender in Research

Cell line source(s)	Human oral epithelial primary cell culture (Celprogen) was used for in vitro evaluation of transfection efficacy of frozen LNP formulation.
Authentication	Primary cell cultures were verified by manufacturer, and published papers. In addition, primary cell cultures were frequently checked by their morphological features. Authentication statements and relevant citations published on the manufacturer's website are listed as follows.<br>Human oral epithelial primary cell culture: https://celprogen.com/human-oral-epithelial-primary-cell-culture-frozen-vial/
Mycoplasma contamination	All cell lines were regularly tested to be mycoplasma-negative by commercial test kit (Lonza).
Commonly misidentified lines (See ICLAC register)	No commonly misidentified cell lines were used in this research.

Animals and other research organisms

Policy information about studies involving animals; ARRIVE guidelines recommended for reporting animal research, and Sex and Gender in Research

Laboratory animals	Yorkshire pigs (Tufts), female, 55-95 kg, 3-5 months old; gouramis (Osphronemidae) (Petsmart), female, 5 cm body length (BL), 5-7 month old; tilapias (Oreochromis) (procured from a local supermarket), 6-9 month old, sex undetermined, 20 cm BL; remoras (Echeneis naucrates) (procured from a local fish store), age and sex undetermined, 10 cm BL
Wild animals	The study did not involved wild animals
Reporting on sex	The primary objective of the studies conducted was to investigate the mechanism of mechanical adhesion, with a specific emphasis on validating MUSAS as a versatile platform for biomedical sensing and drug delivery. Furthermore, the study design aimed to minimize potential confounding factors and sources of variation that could influence the outcomes. A standardized experimental setup was selected to enhance the ability to detect and evaluate the effects of the specific adhesive parameters or intervention under investigation. While recognizing that sex can play a significant role in certain areas of research, the specific study did not involve aspects where sex-specific findings were expected or directly relevant to the research objectives.
Field-collected samples	This research did not include field-collected samples
Ethics oversight	Committee on Animal Care at MIT and the Institutional Animal Care and Use Committee of Boston College

Note that full information on the approval of the study protocol must also be provided in the manuscript.

Plants

Seed stocks	N/A
Novel plant genotypes	N/A
Authentication	N/A
